# Evolution of body size, vision, and biodiversity of coral-associated organisms: evidence from fossil crustaceans in cold-water coral and tropical coral ecosystems

**DOI:** 10.1186/s12862-016-0694-0

**Published:** 2016-06-16

**Authors:** Adiël A. Klompmaker, Sten L. Jakobsen, Bodil W. Lauridsen

**Affiliations:** Florida Museum of Natural History, University of Florida, 1659 Museum Road, PO Box 117800, Gainesville, Florida 32611 USA; Oertijdmuseum De Groene Poort, Bosscheweg 80, NL-5283 WB Boxtel, The Netherlands; Department of Integrative Biology and Museum of Paleontology, University of California, Berkeley, 1005 Valley Life Sciences Building #3140, Berkeley, California 94720 USA; Natural History Museum of Denmark, Geological Museum, University of Copenhagen, Øster Voldgade 5-7, 1350 Copenhagen K, Denmark; Geological Survey of Denmark and Greenland, GEUS, Øster Voldgade 10, 1350 Copenhagen K, Denmark

**Keywords:** Carbonates, Conservation, Corals, Crustacea, Decapoda, Ecology, Ecosystem engineer, Environment, Evolution, Reef

## Abstract

**Background:**

Modern cold-water coral and tropical coral environments harbor a highly diverse and ecologically important macrofauna of crustaceans that face elevated extinction risks due to reef decline. The effect of environmental conditions acting on decapod crustaceans comparing these two habitats is poorly understood today and in deep time. Here, we compare the biodiversity, eye socket height as a proxy for eye size, and body size of decapods in fossil cold-water and tropical reefs that formed prior to human disturbance.

**Results:**

We show that decapod biodiversity is higher in fossil tropical reefs from The Netherlands, Italy, and Spain compared to that of the exceptionally well-preserved Paleocene (Danian) cold-water reef/mound ecosystem from Faxe (Denmark), where decapod diversity is highest in a more heterogeneous, mixed bryozoan-coral habitat instead of in coral and bryozoan-dominated facies. The relatively low diversity at Faxe was not influenced substantially by the preceding Cretaceous/Paleogene extinction event that is not apparent in the standing diversity of decapods in our analyses, or by sampling, preservation, and/or a latitudinal diversity gradient. Instead, the lower availability of food and fewer hiding places for decapods may explain this low diversity. Furthermore, decapods from Faxe are larger than those from tropical waters for half of the comparisons, which may be caused by a lower number of predators, the delayed maturity, and the increased life span of crustaceans in deeper, colder waters. Finally, deep-water specimens of the benthic crab *Caloxanthus* from Faxe exhibit a larger eye socket size compared to congeneric specimens from tropical reefs, suggesting that dim light conditions favored the evolution of relatively large eyes.

**Conclusions:**

The results suggest a strong habitat control on the biodiversity of crustaceans in coral-associated environments and that the diversity difference between deep, cold-water reefs and tropical reefs evolved at least ~63 million years ago. Futhermore, body size and vision in crustaceans evolved in response to environmental conditions in the deep sea. We highlight the usefulness of ancient reefs to study organismal evolution and ecology.

**Electronic supplementary material:**

The online version of this article (doi:10.1186/s12862-016-0694-0) contains supplementary material, which is available to authorized users.

## Background

Although modern cold-water coral ecosystems are much less studied than their warm, shallow-water reef counterparts, they have received considerable attention in the last decade [1–6]. This is in part because these ecosystems are under threat as are tropical reefs and their associated biodiversity [3, 7–11]. The number of reef-building coral species is relatively low for cold-water coral carbonate reefs/mounds sensu [4] in modern and fossil ecosystems [12, 13]. The diversity of associated organisms in modern cold-water habitats might be comparable to the diversity found in tropical coral reefs [5], but the sparse data thus far suggests that diversity is lower in cold-water coral ecosystems [4, 14, 15]. Compared to their direct surroundings, cold-water reefs/mounds are regarded as biodiversity hotspots and the biodiversity of associated organisms is relatively high [4, 5, 12, 16–18], despite their limited areal extent compared to other deep-water habitats [18]. Cold-water coral ecosystems likely represent important speciation centers and glacial refugia in the deep sea [5], can alter hydrography [19], and provide a habitat, food source, and nurseries for various taxa [10, 17]. Thus, they can be considered ecosystem engineers [20, 21].

One of the taxa associated with corals are the highly diverse decapods [15, 22–24], crustaceans that have an extensive fossil record and frequently inhabited tropical reefs in the Meso- and Cenozoic [25–31]. Decapods are usually abundant in and around modern cold-water reefs [15, 16, 24, 32–40], with carid and penaeoid shrimps often comprising the majority of the decapod specimens [33, 36], but true crabs and squat lobsters are also found frequently. This pattern is also reflected in the species composition to some extent (Table 1). Although sampling may not have been exhaustive, species richness is typically < 30 inside or very near to deep-water (200+ m) coral patches, whereas > 30 species are usually found in modern tropical coral reefs (Table 1). The coral framework and the sediments surrounding deep-water corals serve for feeding purposes and as a shelter [17, 33, 34, 36, 39]. Some reports mention that the coral framework contains fewer specimens and species than nearby areas [35, 36]. As is often the case for the number of specimens per taxon, decapod size is rarely recorded with one exception where carid and penaeid shrimp and lobster carapace lengths were compared in- and outside the coral framework [36].

**Table 1:** The number of decapod species found in or around modern coral habitats. The cold, deep-water coral environments overlap in terms of depth with the inferred depth of the Paleocene (Danian) Faxe coral-bearing deposits in Denmark (200–400 m, [13]). For comparison, species richness is also shown for some shallow-water, tropical coral reefs

Closest country/region	Water depth (m)	Number of species	True crabs (Brachyura)	Squat lobsters (Galatheoidea)	Other lobsters	Shrimps (Penaeoidea)	Shrimps (Caridea)	Hermit crabs (Paguroidea)	Other Anomura	Reference
Norway	240–290	3	1	1					1	[32]
Norway (Sula)	275–295	5		1			4			[128]
Italy	350–1100	7	1		2	2	2			[36]
Italy (Sardinia)	380–460	7	4	1			2			[40]
Italy^a^	280–1121	20	7	2	2	3	6			[35]
USA (Alaska)	161–365	2+								[33]
Colombia (Caribbean)	200–220	27	14	1			5	6	1	[129]
Canada	246–630	2								[15]
Panama (Pacific, 3 localities)^b^	2, shallow subtidal	37–55								[69, 130, 131]
Cuba (reef flat, 3 localities)	≤5	36–40								[132]
W Australia (3 localities)^b^	≤2	26–32								[133]
Central Pacific (5 localities)^b^	10	68–191								[134]

^a^large area and depth range, ^b^collecting from corals only

These crustaceans have rarely been studied from fossil cold-water coral ecosystems, in part because such deposits appear less abundant than their warm, shallow-water reef counterparts [41–43]. Usually, decapods from fossil cold-water coral deposits are mentioned only briefly [44–47], except from the Paleocene (middle Danian) of Faxe in Denmark, where they are well-preserved and well-documented taxonomically [48–56].

The mounds at Faxe (or Fakse) are formed predominantly by the frame-building coral *Dendrophyllia candelabrum* [57], with minor occurrences of *Oculina becki* [58] and *Faksephyllia faxoensis* [59]*.* This ancient ecosystem consisted of numerous individual mounds of 50–100 m in diameter, resulting from complex interactions between biological and geological processes [13]. Smaller bryozoan mounds and intervals with an octocoral-rich facies are interfingering with the larger coral mounds [60]. The corals grew in relatively deep water below the photic zone, between 200–400 m [13] in the mesopelagic zone. At such depths, only some light penetrates so that animals may still be able to detect objects against downwelling light [61]. A variety of associated organisms is found at Faxe including annelids, arthropods, brachiopods, bryozoans, echinoderms, and mollusks [51, 60, 62–64].

Faxe yields the best known fossil decapod fauna from a cold-water coral ecosystem by far [51], but decapod diversity differences within this varied environment have not been investigated. Furthermore, this unique cold-water fauna has not been compared to tropical counterparts in terms of biodiversity, body size, and eye size. Consequently, the effect of environmental conditions on decapod faunas in these two environments is not well-known. The biodiversity, body size, and eye size of these crustaceans is not compared extensively for today’s cold-water coral and tropical coral environments, at least in part due to the cryptic nature of crustaceans in modern coral ecosystems, hampering unbiased collecting and comparisons. Such studies on extant coral habitats will get increasingly difficult due to coral decline worldwide [3, 7–11]. Here we use fossil decapods from coral-associated habitats that formed and ultimately vanished prior to human disturbance to test the following hypotheses:

1. Decapod diversity is higher in the coral facies compared to that from the intercalated bryozoan facies at Faxe.
2. Decapod biodiversity is higher in warm, shallow-water reefs compared to that from the cold-water mounds at Faxe.
3. Decapods from cold-water coral reefs/mounds are larger than those from shallow-water reefs, probably due to the lower number of predators in deeper waters and/or because of the physiology of crustaceans in deeper, relatively cold waters resulting in a delayed maturity and an increased life span.
4. Deeper water specimens from Faxe exhibit a relatively large eye socket size as an adaptation to increase light capture of downwelling and bioluminescent sources.

We show differences in decapod biodiversity within the Faxe mound ecosystem and among the cold-water reef/mounds at Faxe versus those from other European reefs. Carapace size differs significantly in part of the analyses comparing Faxe decapods to other assemblages. Finally, we report on significant differences in eye socket size of the benthic crab *Caloxanthus* from a deep-water setting compared to a shallower environment.

## Methods

### Sampling

**Fig. 1:**
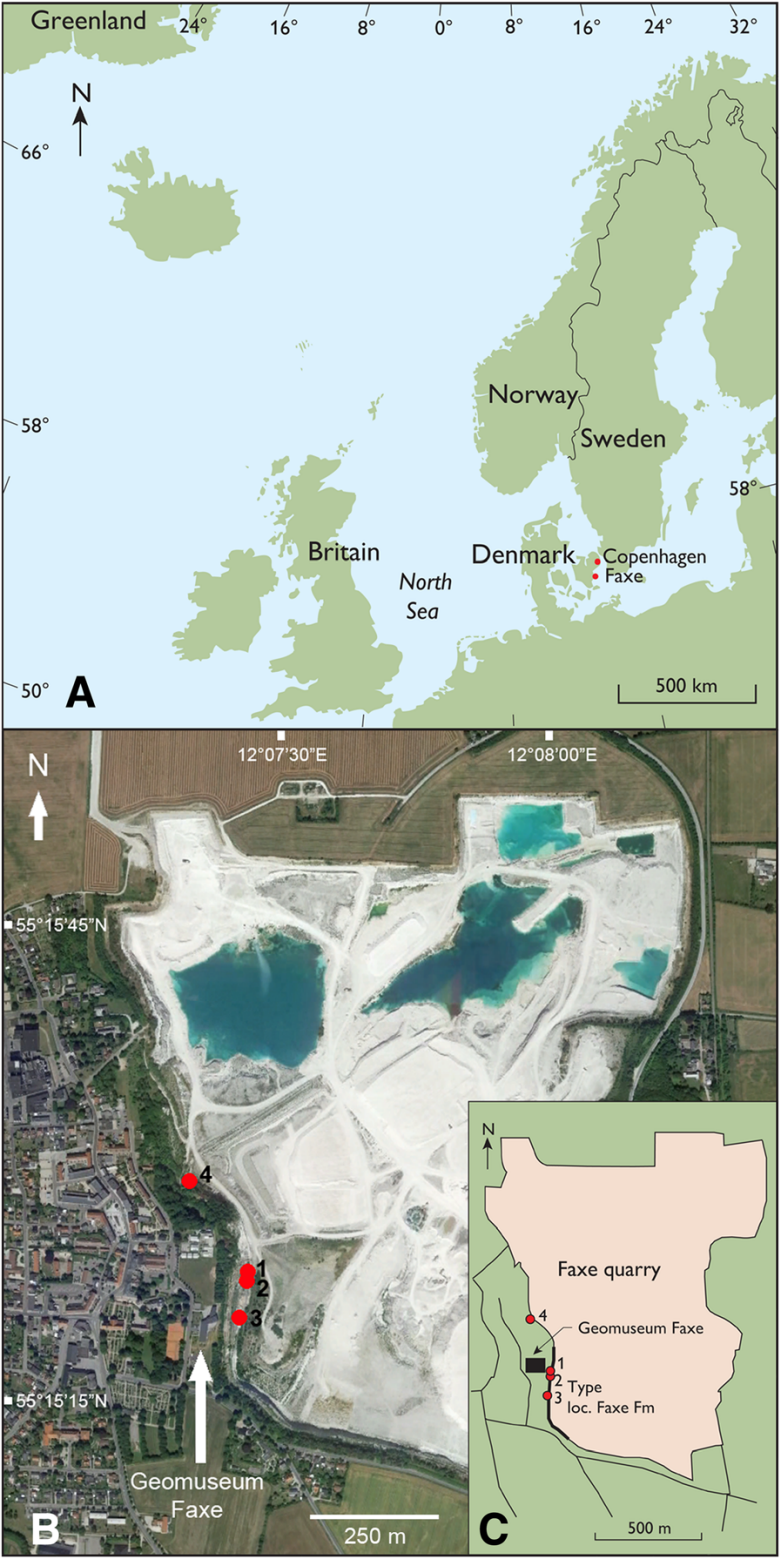
The location of Faxe in Denmark in Europe and an overview of the Faxe Quarry. **a** Faxe in Europe. **b**, **c** The location of the four sample sites. Google Earth image (20 July 2013) of the Faxe Quarry, Sjælland, Denmark

Sampling took place at four sites in the Faxe Formation in the Faxe Quarry, Denmark [13, 60], on the southwestern wall directly below and slightly north of the Geomuseum Faxe (55°15’20”N/12°07’26”E – 55°15’29”N/12°07’21”E) (Fig. 1). Four *in situ* samples per site were extracted from a few square meters by filling a bucket of 22,850 cm^3^ for each sample. Volume instead of collecting time [29] was chosen here because of marked differences in hardness of the rock among the sites (more volume would be quarried for softer rocks in an equal amount of time). The rocks were broken into subequal pieces of a few cm^3^ subsequently and all decapods were collected. An approximately equal amount of time per site was spent in the sunshine because decapods may be observed more readily in the sun [29]. For all statistical analysis PAST 3.06 [65] and a significance level of 5 % was used.

**Fig. 2:**
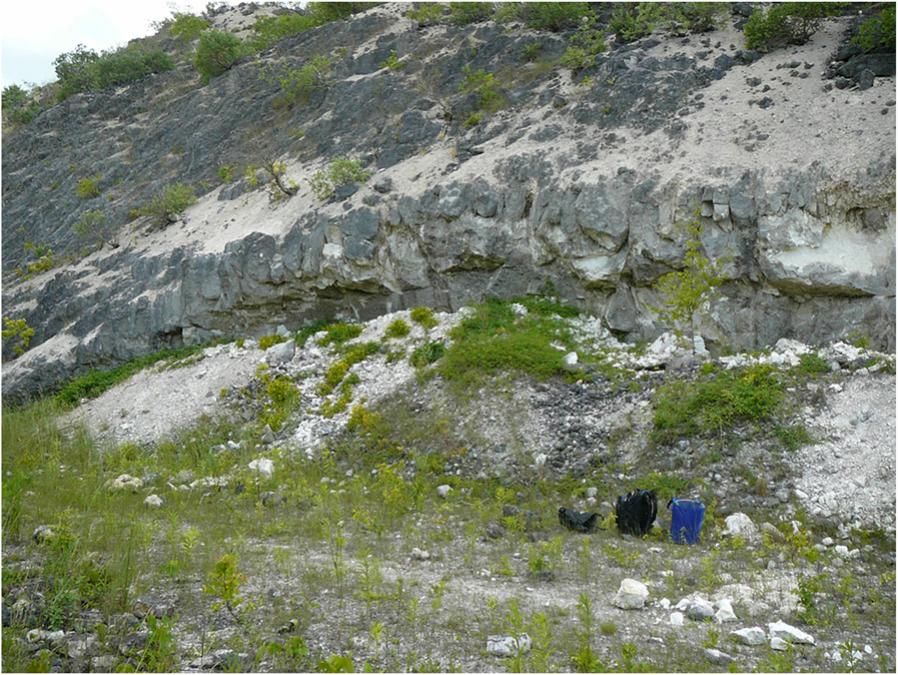
Dome-shaped octocoral site (site 1) surrounded by scleractinian coral bafflestones. Sampling took place to the right of the small tree above the bucket (blue) at the level of the tree (see also Additional file 1: Figure S1). The site with the coral bafflestones (site 2) is located towards the left of the dome (not visible here). The bucket is 34 cm tall

The four sample sites expose different facies. The samples collected directly west of (below) the Geomuseum Faxe consist of dense bryozoan-dominated rudstone with rare remains of corals, brachiopods, decapods, and bivalves (site 3). Over 80 % of the limestone consists of bryozoa. The scleractinian coral dominated site (site 2), slightly to the north of site 3 and adjacent to the ‘octocoral’ site, consists of a dense network of branching coral bafflestone (Fig. 2). It consists predominantly of branches and colony fragments of the mound-forming coral *Dendrophyllia candelabrum*, often only some centimeters apart, in between which some brachiopods, gastropods, bivalves, and decapods are found. The octocoral packstone site (site 1), containing octocorals, scleractinians, and bryozoans primarily, is part of a dome-shaped structure surrounded by coral bafflestones. Other associated fossils are brachiopods, gastropods, bivalves, and decapods. These three sites are part of the type locality of the Faxe Formation [60]. The northernmost bafflestone site (site 4) is composed of intermixed bryozoans and scleractinian corals, not as densely packed as the second site, but with many pockets filled with carbonate mud and abundant decapods as well as brachiopods, gastropods, and bivalves.

### Diversity

For counting the number of specimens per species, a number of standardizations were carried out to minimize biases (see [29], for further discussion): (1) only internal molds (with or without cuticle) of carapaces with the axial part of the cervical groove preserved (a unique landmark) were counted; (2) specimens were collected by AAK only to minimize the possible effect of different sample strategies.

To investigate diversity differences among sites within the quarry, multiple measures of diversity were employed and all samples per site were merged for an adequate sample size for comparisons among sites. All specimens determined to the species-level were included in the analyses. However, 13–22 % of the specimens per site could not be determined to the species-level due to their inadequate preservation, but only to the genus to superfamily-level. These specimens were assigned to species based on the relative proportion of species of the respective taxon at each site to increase sample size (Additional file 1: Table S1). Diversity per site was computed using: (1) species and genus richness, (2) individual rarefaction per site, and (3) the Shannon Index.

Decapod diversity of fossil cold, deep and warm, shallow-water coral-associated reefs sensu [66] was compared using the number of species and genera per locality for various localities in Europe (Faxe Quarry, Paleocene, Danian, Denmark; ENCI Quarry/St. Pietersberg, Upper Cretaceous, Maastrichtian, The Netherlands; Koskobilo Quarry, mid-Cretaceous, Albian, Spain; Contrada Gecchelina di Monte di Malo, lower Eocene, Ypresian, Italy; Braggi Quarry at Vestenanova, lower Eocene, Ypresian, Italy). All localities have been adequately sampled so that most species have been collected, they are found on the same continent, and they are exposing sediments with a minimum possible age range for suitable localities (~50 Myr). Abundance data per species is unavailable for part of the localities, but they are for the Koskobilo and Faxe quarries so that diversity analyses (2) and (3) as above could be performed. All available specimens used here were systematically collected by AAK at both localities and samples were merged to increase sample size for all sites per locality.

### Body size

The maximum carapace width of decapods was measured for each possible specimen collected in Faxe (measurements as in [29]). Width was chosen over length because width could be measured more easily for partial specimens due to the bilateral symmetry of decapods. The non-parametric Kruskal-Wallis test was used to compare widths among sites from the Faxe Quarry. The Mann–Whitney pairwise comparisons test (with and without a Bonferroni correction) was used to assess whether pairs differed significantly. The same was done for the two most abundant species: *Dromiopsis elegans* [67] and *D. rugosus* [68]. Carapace widths were also compared between Koskobilo (data from [29]) and Faxe using the Mann–Whitney test for all available data and for similar coral facies only (site 17 at Koskobilo and sites 2 + 4 at Faxe) because decapod size may change per habitat [69], even within reefs [29].

Carapace widths (and geometric means where possible) of decapods and a subset (true crabs or Brachyura, the most speciose decapod clade) from Faxe were also compared, using the Mann–Whitney test, to those from the other localities with coral limestones as mentioned above. For this purpose, the maximum carapace width and length were recorded as derived from the literature or as measured herein. Furthermore, the maximum widths of crab genera abundantly present in both Faxe and Koskobilo (*Caloxanthus* and *Faksecarcinus*) were also compared. Also, the maximum widths of *Dromiopsis praelaevior* [70] from the ENCI Quarry and *D. paucigranosa* [27] from Contrada Gecchelina di Monte di Malo and the Braggi Quarry were compared to those of congenerics from Faxe. *Dromiopsis mosae* [70] was recently suggested not to originate from the ENCI Quarry [71] and was not used. As Brachyura are the most speciose decapod clade known from the Paleocene, the maximum widths and geometric means of reef-associated crabs from Faxe were compared to those of all other Paleocene crabs.

### Eye socket size

Eye sockets (or orbits) are preserved for some decapod taxa and serve as a proxy for eye size. Eye socket height is measured here because socket width is frequently influenced by the eye stalks as well. As eye size is correlated with specimen size, carapace widths and lengths were also measured to obtain the geometric mean of the carapace. Differences in eye size between deep and shallow-water decapods are best expressed by comparing taxa within the same genus [72]. For that reason, eye socket heights of congeners from different inferred paleodepths are compared. The epifaunal crabs of *Caloxanthus* were used for this purpose because they are abundant in Faxe (*Caloxanthus ornatus* [48]) and are also commonly found in shallow-water coral-associated rocks from the Albian of Koskobilo (*C. paraornatus* [73]); a single specimen is reported from the Danian of Vigny (*C. vignyensis* [74]). No other genera were suitable for this purpose.

Specimens of museum collections were studied to obtain eye socket height and carapace size of *Caloxanthus*. Specimens of comparable width (≤ 6.5 mm) and geometric size (≤ 5.6) of *Caloxanthus* from Faxe and Koskobilo with a comparable sample size were used for a one-way ANCOVA analysis to compare the adjusted means and slopes. Using specimens of similar carapace size is important because eye size tends to scale with body size, but the performance of eyes (e.g., spatial resolution and sensitivity) improves as absolute size increases [61].

## Results

### Diversity and taphonomy sample sites at Faxe

**Fig. 3:**
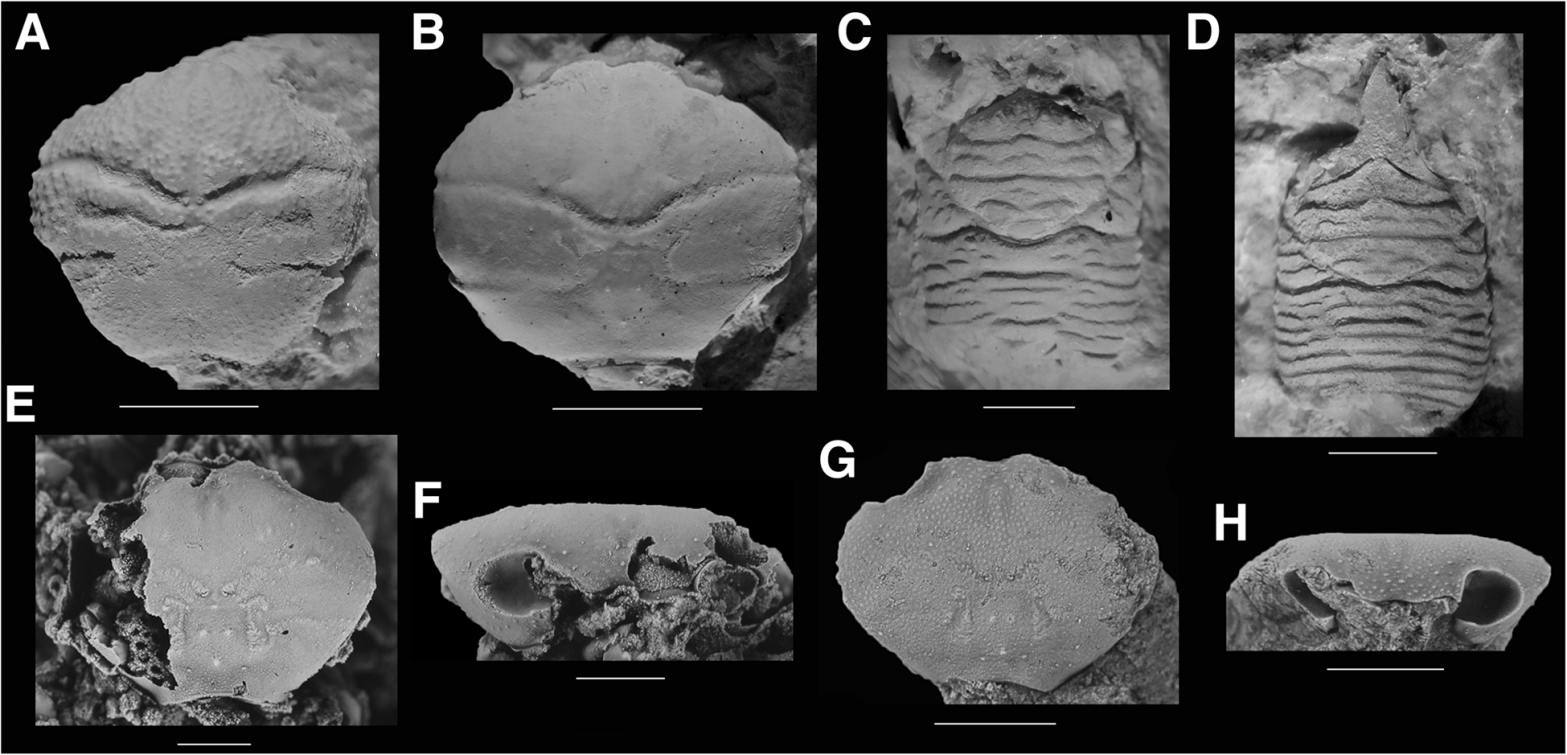
Carapaces of common decapod species from Faxe and one from Koskobilo. Carapaces from the Paleocene (middle Danian) of Faxe, Denmark (**a**–**f**), and a carapace from the Albian of Koskobilo, Spain (**g**–**h**), for comparison. **a** *Dromiopsis rugosus* (UF 256316). **b** *Dromiopsis elegans* (UF 256314)*.* **c** *Protomunida munidoides* (UF 256317). **d** *Galathea strigifera* (UF 256315)*.* **e**, **f** *Caloxanthus ornatus* (dorsal and frontal view showing right orbit, MAB k3153). **g**, **h** *Caloxanthus paraornatus* (dorsal and frontal view showing left orbit, MGSB 77703, holotype). Permission to use **e**–**h** [73] by The Palaeontological Association. Scale bar width: 5.0 mm for A–B; 2.0 mm for rest.

**Fig. 4:**
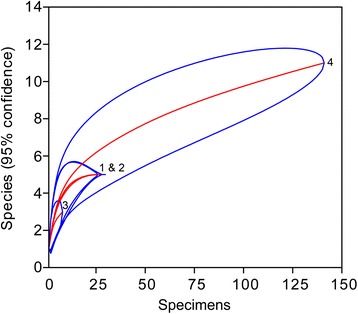
Rarefaction curves for decapods of the four sites with 95 % confidence intervals. The slopes of the means (middle lines) suggest that site 4 exhibits the highest diversity

**Fig. 5:**
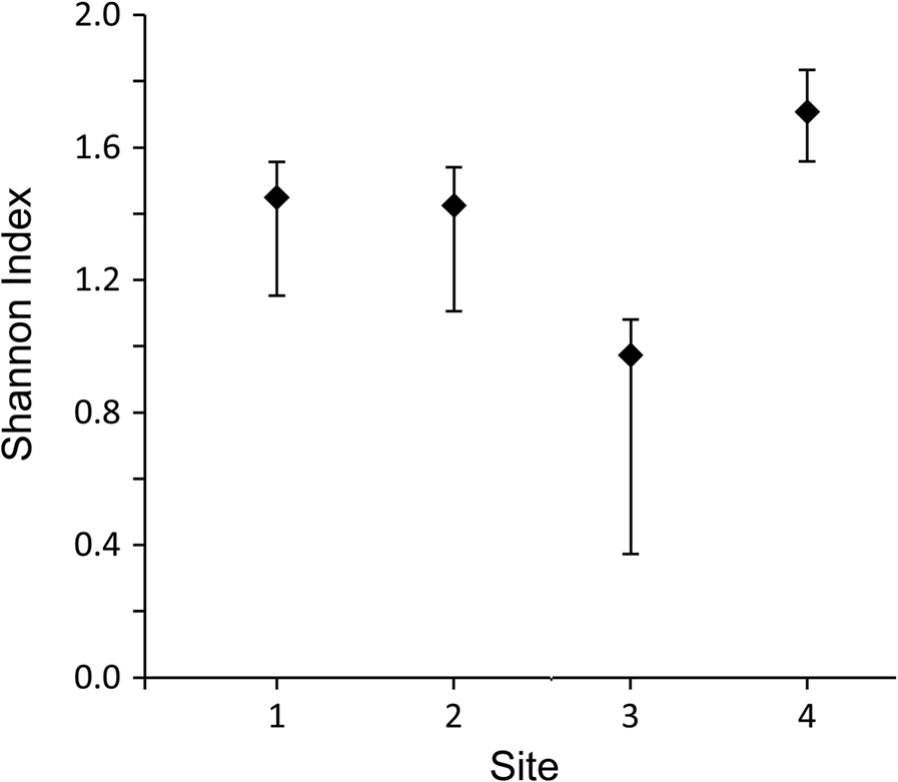
Shannon index for decapods of the four sites with 95 % confidence intervals (bootstrapped, 9999 random samples)

Species that are almost always found at each site are *Dromiopsis rugosus*, *D. elegans*, *Protomunida munidoides* [49], *Galathea strigifera* [48], and *Caloxanthus ornatus* (Fig. 3; Additional file 1: Table S1). *Dromiopsis elegans* is the most common species at each site, except for site 2 where *D. rugosus* is represented by one more specimen. Species and genus richness (Table 2), rarefaction analyses (Fig. 4), and the Shannon Index (Fig. 5) all suggest that site 4 is most diverse. These results were replicated when specimens not determined to the species-level were excluded.

**Table 2:** Decapod abundance and taxon richness per site

	site 1 (octocoral packstone)	site 2 (bryozoan rudstone)	site 3 (scleractinian coral bafflestone)	site 4 (scleractinian coral-bryozoan bafflestone)
# specimens	27	32	9	141
# species	5	5	3	11
# genera	4	4	3	9

Rocks at all sites were lithified, but the carbonates at sites 1 and 3 were softer and more friable than other sites. This did not affect the recognition of decapods substantially, with the possible exception of site 1 where decapods were less well-preserved. All specimens used consist of complete or partial dorsal carapaces without venters and appendages attached. Cuticle is preserved occasionally, but this did not influence the identification of specimens to the species-level here [74] because the taxonomy of decapods from Faxe is well-known [51, 55]. Shrimps, abundant in modern cold-water corals habitats (Table 1), were absent.

### Diversity Faxe vs other European coral-associated localities

**Fig. 6:**
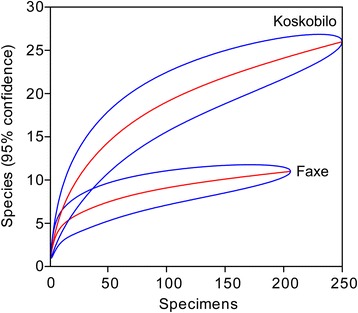
Rarefaction curves for decapods of Faxe and Koskobilo with 95 % confidence intervals. Both localities incorporate various microhabitats. Data for Koskobilo from [29]

Species richness comparisons indicate that both the number of decapod genera and species is lowest at Faxe (Table 3), and rarefaction analyses show a lower diversity at Faxe compared to Koskobilo (Fig. 6). The Shannon Index of Faxe (1.71; 95 % confidence interval: 1.60–1.82) is also lower than that of Koskobilo (2.42; 2.30–2.56).

**Table 3:** Decapod species and genus richness per locality. Faxe is the only locality associated with cold-water corals; the rest is associated with warm, shallow-water associated corals

	Faxe, Denmark (Paleocene, Danian)	ENCI/St. Pietersberg, The Netherlands (Upper Cretaceous, Maastrichtian)	Koskobilo, Spain (mid-Cretaceous, Albian)	Contrada Gecchelina di Monte di Malo, Italy (Eocene, Ypresian)	Braggi Quarry at Vestenanova, Italy (Eocene, Ypresian)
# species	25	29	38	48	46
# genera	18	27	27	42	36

Sources used: Faxe [51, 55, 135]; ENCI/St. Pietersberg ([70, 71, 136–142], RHB Fraaije and JWM Jagt, personal communication 2015); Koskobilo [73, 88, 143–149]; Contrada Gecchelina di Monte di Malo [27]; Braggi Quarry [31]

### Body size

Decapod widths per site at Faxe differ significantly (Kruskal-Wallis *H* = 15.2, *p* = 0.002) (data: Additional file 1: Table S2). After a Bonferroni correction, only the median sizes of sites 2 and 4 differ significantly using the Mann–Whitney test (medians 7.56 and 4.46 mm, resp.) (Table 4). Using only the two most abundant species yielded no significant differences (*Dromiopsis elegans*: Kruskal-Wallis *H* = 7.5, *p* = 0.06; *D. rugosus*: Kruskal-Wallis *H* = 4.7, *p* = 0.10).

**Table 4:** *P*-values of the Mann–Whitney tests on decapod widths of the four sites at Faxe

	site 1 (octocoral packstone)	site 2 (bryozoan rudstone)	site 3 (scleractinian coral bafflestone)	site 4 (scleractinian coral-bryozoan bafflestone)
site 1	-	0.3362	0.3205	0.0301*
site 2	1	-	0.0384*	0.0005*
site 3	1	0.2301	-	0.9615
site 4	0.1803	0.0031*	1	-

Without (upper right) and with (lower left) Bonferroni correction applied; statistically significant results indicated by *

Comparing all decapod size data based on collected specimens from all sites at Faxe and Koskobilo yielded no significant difference (Mann–Whitney *U* = 0.0002, *p* = 0.89). However, when decapods from comparable facies (coral boundstones) were analyzed, decapods from Faxe were larger than those of Koskobilo (Mann–Whitney *U* = 5322, *p* = 0.006; medians 4.8 vs 4.1 mm, resp.).

Comparing the maximum sizes of decapod species from Faxe to those from other coral-associated localities (Additional file 1: Tables S3–7) showed that part of the assemblages were statistically indistinguishable, whereas Faxe decapods were larger in half of the tests (Table 5). A larger size is also observed for the benthic crab *Caloxanthus*: maximum width for the species from Faxe is 13.8 mm (*n* = 85), whereas the maximum width is 6.6 mm (*n* = 27) for Koskobilo (Additional file 1: Table S8). A Mann–Whitney test on *Caloxanthus* specimens of both samples shows statistical difference between the medians (*U* = 221.5, *p* < 0.00001). The single specimen of *Caloxanthus* known from the shallow-water, coral-associated Danian of Vigny [74] is also smaller (6.5 mm) than the maximum size of *Caloxanthus* from Faxe. Conversely, the maximum width of the swimming crab *Faksecarcinus* appears larger in Koskobilo (17.5 mm, *n* = 9) compared to Faxe (13.6 mm, *n* = 16) (Additional file 1: Table S8), but the medians are not statistically significant (Mann–Whitney *U* = 44, *p* = 0.12). Although *Dromiopsis praelaevior* from the ENCI Quarry is only known from a single specimen [70], it exhibits a smaller maximum width compared to all four congenerics from Faxe (Additional file 1: Tables S3–7). Likewise, specimens of *Dromiopsis paucigranosa* from Contrada Gecchelina di Monte di Malo and the Braggi Quarry [27, 31] are smaller than *Dromiopsis* spp. from Faxe (Additional file 1: Tables S3–7).

**Table 5:** *P*-values of Mann–Whitney tests comparing decapod sizes of Faxe to those of various other localities

	ENCI/St. Pietersberg, The Netherlands (Upper Cretaceous, Maastrichtian)	Koskobilo, Spain (mid-Cretaceous, Albian)	Contrada Gecchelina di Monte di Malo, Italy (Eocene, Ypresian)	Braggi Quarry at Vestenanova, Italy (Eocene, Ypresian)
Faxe (all decapods) - width	0.54	0.01*	0.70	0.13
Faxe (all crabs) - width	0.14	0.01*	0.69	0.01*
Faxe (all decapods) - geometric mean	0.32	0.05*	-	-
Faxe (all crabs) - geometric mean	0.05*	0.03*	-	-

Statistically significant results indicated by *, all of which imply that Faxe decapods are larger. Length was infrequently available for taxa from the Italian localities; hence, no geometric means were used for comparisons

Finally, the width and geometric means of Faxe crabs and all other Paleocene crabs (Additional file 1: Table S9) do not differ significantly (Mann–Whitney *U*-values: 453, 432; *p*-values: 0.62, 0.72, resp.).

### Eye socket size

**Fig. 7:**
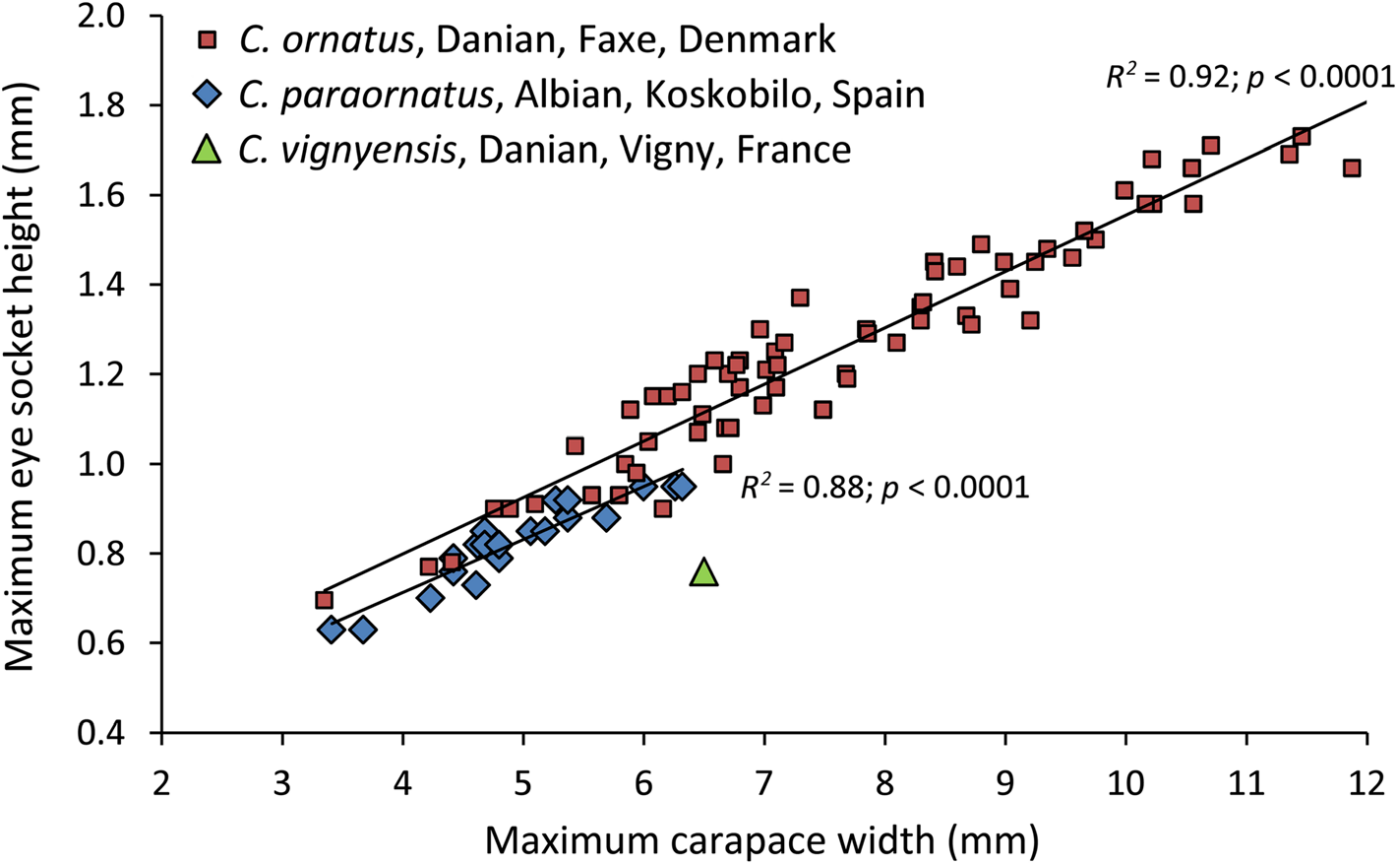
Eye socket height of three species of the crab *Caloxanthus* vs the maximum carapace width

The trend line of eye socket height versus maximum carapace width of *C. paraornatus* plots below that of *C. ornatus* (Fig. 7; Additional file 1: Table S8). A one-way ANCOVA test suggests that the slopes do not differ (*F* = 1.46, *p* = 0.23), but the adjusted means do (*F* = 17.34, *p* = 0.0002), indicating that eye socket height is significantly greater for similar-sized specimens from Faxe. Similar results are obtained when using the geometric mean (*F* = 2.10, *p* = 0.16; *F* = 8.77, *p* = 0.006, resp.). The eye socket height of the single specimen of *C. vignyensis* is smaller relative to the other species for the same carapace width.

## Discussion

### Diversity and taphonomy sample sites at Faxe

The hypothesis that decapod diversity is higher in coral facies compared to that of the intercalated bryozoan facies is partially supported. The coral-dominated facies (site 2) harbors a higher diversity compared to the bryozoan-dominated facies (site 3) (Figs. 4 and 5; Table 2). This is consistent with the suggestion that decapod diversity in Eocene bryozoan-associated limestones from the USA (South Carolina) was lower than in coral-associated limestones in Europe, primarily due to ecological differences [75]. Although modern bryozoan habitats also contain a diverse, mostly facultative associated fauna including decapods [76–79], the relative diversity of this fauna compared that of coral reefs is unknown. At Faxe, decapod diversity and abundance is highest in the facies dominated by a mixture of bryozoans and scleractinian corals. This result cannot be explained by preservational differences because the coral-dominated site is composed of bafflestones of about equal hardness, whereas the bryozoan-dominated site is slightly softer, but decapod preservation is largely similar. The extensive, dense network of monotonous branching corals of primarily *Dendrophyllia candelabrum* at site 2 (only some centimeters between each branch) probably precluded inhabitation of an abundant, diverse decapod fauna, whereas a more varied, open framework with a higher number of microhabitats at site 4 attracted more decapods. This result may be consistent with data from modern cold-water coral habitats because: (1) the coral framework itself seems to harbor a relatively low diversity [7, 35, 36] and (2) habitat heterogeneity appears to promote diversity [34, 69, 80]. The difference in abundance between sites 2 and 4 is partly caused by galatheoids that make up 54 % of the decapod fauna at site 4, whereas squat lobsters are less abundant at other sites (≤ 33 %). Preservation could have played a role for site 1, where sediments are much softer and decapod preservation is less pristine regardless of carapace size compared to other sites. Previous work showed that decapod diversity and abundance was highest in a coral bafflestone from the mid-Cretaceous (Albian) of Spain [29] compared to more open habitats, also partially caused by abundant galatheoids (51 %) compared to two out of three other sites. As for many modern cold-water coral faunas (Table 1), true crabs and squat lobsters are common. Shrimps are most likely absent because they do not preserve well [81, 82].

### Diversity Faxe vs other European coral-associated localities

The relatively low decapod diversity of Faxe compared to that of other European, Cretaceous–Paleogene localities associated with corals (Fig. 6; Table 3) can be explained by the environment in which these crustaceans lived. Whereas Faxe corals and the associated organisms inhabited in a deep, cold-water environment (200–400 m, [13]), other coral-associated limestones represent warm, shallow-water deposits as evidenced by the presence of, for example, algae [27, 29, 31, 83]. This low diversity of decapods at Faxe is consistent with the relatively low number of reef-building coral species in modern cold-water reefs [12] and the relatively low fish species richness [4] compared to tropical reefs. Although the data are limited thus far, corals of the deep also harbor a lower species diversity of gastropods, copepods, and decapods compared to their tropical counterparts (Table 1) [14, 15, 84].

**Fig. 8:**
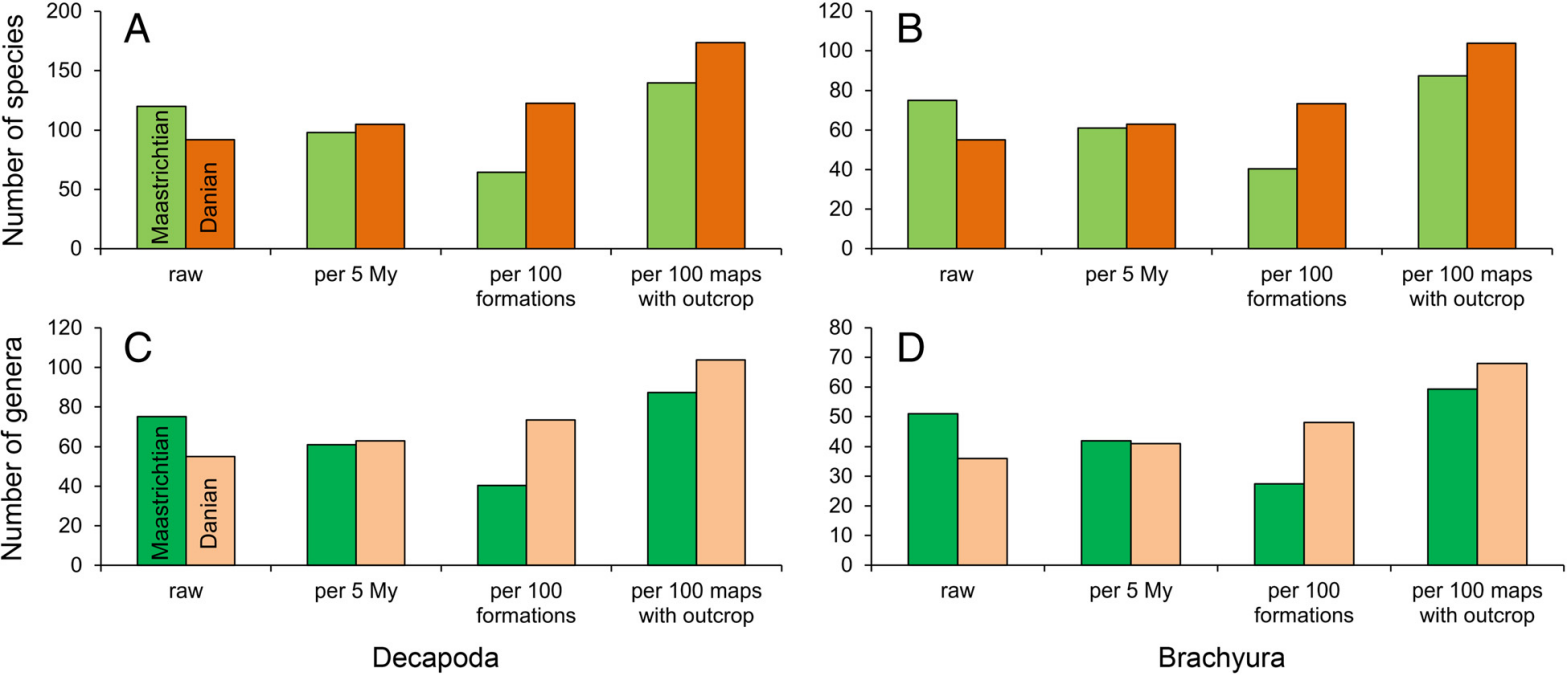
Standing global diversity of decapods and Brachyura or true crabs across the Cretaceous/Paleogene boundary. Diversity is shown at the species- and genus-levels. Four sets of histograms are used to compare the number of taxa present in the Maastrichtian and the Danian: raw, standardized per 5 my, standardized per 100 marine formations globally as a proxy for outcrop area, and standardized per 100 maps with marine outcrops in Western Europe as a proxy for outcrop area [127]. **a** Decapod species. **b** Brachyuran species. **c** Decapod genera. **d** Brachyuran genera

Alternative explanations for this difference in biodiversity are (1) a lower sampling intensity for Faxe; (2) the fact that the Faxe fauna lived shortly after the Cretaceous/Paleogene extinction event [60, 85–87], which may have impacted decapod diversity as well; (3) a latitudinal signal; and (4) a lower preservation potential of decapods at Faxe. A lower sampling intensity is easily rejected because Faxe has been sampled extensively as > 5000 decapod specimens are known [51], which is more than, for example, Koskobilo [88]. Moreover, the rarefaction trajectories also suggest a lower diversity for Faxe compared to Koskobilo (Fig. 6). Previous work has suggested that the Cretaceous/Paleogene extinction event appears not to have severely impacted decapod diversity. Seventy-nine percent of decapod families survived into the Paleogene, and many genera were able to survive in the (sub)tropical Americas, relatively close to the Chicxulub impact site [89]. Brachyurans also appear hardly affected [90]. More regionally, 66 % of the Danian crab genera in the Denmark – Sweden region were Cretaceous survivors [50]. Our new analyses of the uncorrected global standing decapod diversity show lower numbers of species and genera in the Danian than in the Maastrichtian. On the species-level, 120 decapod and 75 brachyuran species from the Maastrichtian are known [30], whereas 92 decapod and 55 brachyuran species have been reported from the subsequent Danian (e.g., [89, 91–95]) (Additional file 1: Tables S9–S10). Seventy-five decapod genera including 51 brachyurans are reported from the Maastrichtian, whereas 55 decapod genera including 36 crabs are known from the Danian. However, when adjusted for the unequal duration of both stages and the differences in rock outcrop area (Fig. 8; Additional file 1: Tables S10–11), the standing diversity does not drop for both taxonomic levels. Instead, Danian diversity is either approximately equal to that of the Maastrichtian or higher in the Danian. Unlike for many other organisms worldwide [86], these results suggest a limited signal of a mass extinction in decapods and true crabs across this interval. Another factor that may have influenced the patterns is latitude because modern decapod diversity increases towards the equator [96, 97]. However, all localities addressed herein are within a limited latitudinal range (~40–55°N) consistently throughout the Cretaceous–Paleogene, and the geographically (and temporally) closest locality to Faxe, the ENCI Quarry, is only 4.5° south of Faxe. The decapod carapaces at Faxe are generally well-preserved, also in comparison to, for example, Koskobilo (pers. obs. AAK). In conclusion, the alternatives cannot fully explain to the relatively low decapod diversity in Faxe.

More likely, the pattern can be explained ecologically, consistent with observations from today’s oceans that decapod diversity is lower in deep-water coral habitats (Table 1). Compared to warm, shallow-water reefs, corals at Faxe grew probably slower, likely yielding less food for mucus-feeding decapods and fewer places to hide. Additionally, less coral rubble is produced for these crustaceans to use as a habitat (decapods can also be common in this coral-derived habitat: [34, 36, 39, 69]). The results further imply that the decapod biodiversity differences between deep, cold-water reefs and tropical reefs evolved at least ~63 million years ago, the age of the Faxe deposits [60].

### Body size

The fact that little size variation of decapods exists at Faxe (Tables 4, Additional file 1: Table S2) suggests limited environmental control on body size for the four studied sites. Only sizes at site 4 are significantly smaller than those from site 2. This difference is explained by the relatively high proportion of the small galatheoids at site 4 in combination with the relatively large specimens of *D. rugosus* at site 2.

The hypothesis that decapods in cold waters are larger is mostly supported: decapods from deep, cold waters at Faxe are larger than those of Koskobilo when comparable facies are analyzed; half of the tests comparing decapod sizes of Faxe to the congeners of shallower-water faunas return the same results, whereas the other tests showed no significant difference; sizes of *Faksecarcinus* are not statistically different for Faxe and Koskobilo; *Caloxanthus* from Faxe is larger than those from Koskobilo; and *Dromiopsis* spp. from Faxe is larger than *Dromiopsis* spp. from the ENCI Quarry and both Italian localities. The results are consistent with larger body sizes of various modern crustaceans including decapods at high latitudes and deeper, colder waters [96, 98–102]. An explanation for the evolution of large body size within genera and for assemblages may be related to crustacean physiology because crustaceans in deeper, colder waters experience delayed maturity, possess larger cells, and have an increased life span [100, 102, 103]. High latitudes also promote fewer and larger eggs for various crustaceans so as to increase fecundity, released during a period of optimum conditions [96, 104, 105]. Additionally, a lower impact of predators (e.g., fish) in cold-water reefs/mounds may also have contributed to larger crustaceans [106, 107]. A relatively low fish species richness is observed in modern cold-water reefs/mounds compared to tropical reefs [4], and fish species richness and abundance may be positively correlated [108, 109].

Interestingly, crab width from Faxe does not differ significantly from other Paleocene decapods (mostly from other habitats), but coral-associated decapods from the Cretaceous are smaller than those from other habitats [110]. These Cretaceous decapods are all associated with warm, shallow water coral habitats, in which a large size is not favored [110], whereas decapods from Faxe lived in colder, deeper waters. Hence, a larger size would be expected for these decapods.

### Eye socket size

The hypothesis that deeper water specimens from Faxe exhibit a relatively larger eye socket size as an adaptation to increase light capture of downwelling and bioluminescent sources is supported. Eye socket heights of the benthic crab *Caloxanthus* from Faxe are significantly larger than those of Koskobilo. Moreover, a single specimen from the same geological stage as the Faxe deposits, from the shallow-water reef of Vigny [111, 112], exhibits a smaller eye socket compared to *Caloxanthus* from Faxe. These results are consistent with results from modern, congeneric benthic decapods: deep-water animals including decapods often have relatively large eyes to better detect objects in their dim environment [61, 72, 113]. Conversely, modern deeper water pelagic crustaceans appear to have smaller eyes resulting in a lower energetic burden, a lower weight, less drag, and eyes being less visible to predators [114, 115], a result not studied in fossil decapods thus far. This study shows that fossil decapod crustaceans can be used to study vision and ecology, which has rarely been documented thus far [116, 117] given the rare occurrence of fossil decapod eyes with the exception of specimens from Konservat-Lagerstätten [118–126].

## Conclusions

1. A comparison of decapod crustaceans at different sites at the Paleocene cold-water reef/mound ecosystem from Faxe (Denmark) shows that diversity is highest at the coral-bryozoan dominated site, followed by the coral dominated site and the bryozoan-dominated site.
2. In agreement with the hypothesis, decapod biodiversity is higher in several fossil tropical reefs from Europe compared to that of Faxe using a variety of metrics. Thus, diversity differences between deep, cold-water reefs and tropical reefs evolved at least ~63 million years ago.
3. Decapods from Faxe are larger than those from warm, shallow waters from other localities for half of the analyses carried out, whereas the other half did not show significant differences. These results are in partial agreement with the hypothesis that decapods evolved larger sizes in cold-water coral reefs/mounds because of a lower number of predators, the delayed maturity, and the increased life span of crustaceans in deeper, colder waters.
4. In agreement with the hypothesis, deep-water specimens of the benthic crab *Caloxanthus* from Faxe exhibit a larger eye socket size compared to congeneric specimens from tropical reefs, suggesting that dim light conditions favored the evolution of relatively large eyes.
5. As shown by the body size and eye socket analyses, the effect of environmental conditions acting on the associated organisms of cold-water coral reefs/mounds vs those in tropical reefs are clearly expressed in the morphology of decapod crustaceans.

## Abbreviations

Specimens used in this study are housed in the Florida Museum of Natural History at the University of Florida (UF), Gainesville, Florida, USA; MAB k, Oertijdmuseum De Groene Poort, Boxtel, The Netherlands; MGSB, Museo Geológico del Seminario de Barcelona, Barcelona, Spain; MGUH, GM, Natural History Museum of Denmark, Geological Museum, University of Copenhagen, Denmark; OESM, Geomuseum Faxe, Faxe, Denmark; SNSB-BSPG, Bayerische Staatssammlung für Paläontologie und historische Geologie, München (Munich), Germany

## Additional file

Additional file 1: Figure S1. Collecting sites Faxe. **Table S1**. Abundance data Faxe. **Table S2**. Size data Faxe. **Table S3**. Maximum size data Faxe. **Table S4**. Maximum size data ENCI/St. Pietersberg. **Table S5**. Maximum size data Koskobilo. **Table S6**. Maximum size data Contrada Gecchelina di Monte di Malo. **Table S7**. Maximum size data Braggi. **Table S8**. Carapace and eye socket size measurements. **Table S9**. Maximum size data of Paleocene crabs. **Table S10**. Maastrichtian and Danian decapod diversities. **Table S11**. Data used for Table S10. (DOCX 917 kb)
