# Supplementary material for: Evolution of body size, vision, and biodiversity of coral-associated organisms: evidence from fossil crustaceans in cold-water coral and tropical coral ecosystems
Source: BMC Evol Biol. 2016 Jun 16;16:132. doi: 10.1186/s12862-016-0694-0 (PMC4910220; doi:10.1186/s12862-016-0694-0)
Supplement: Additional file 1: Figure S1. — Collecting sites Faxe. Table S1. Abundance data Faxe. Table S2. Size data Faxe. Table S3. Maximum size data Faxe. Table S4. Maximum size data ENCI/St. Pietersberg. Table S5. Maximum size data Koskobilo. Table S6. Maximum size data Contrada Gecchelina di Monte di Malo. Table S7. Maximum size data Braggi. Table S8. Carapace and eye socket size measurements. Table S9. Maximum size data of Paleocene crabs. Table S10. Maastrichtian and Danian decapod diversities. Table S11. Data used for Table S10. (DOCX 917 kb) [file 12862_2016_694_MOESM1_ESM.docx]

**Evolution of body size, vision, and biodiversity of coral-associated organisms: evidence from fossil crustaceans in cold-water coral and tropical coral ecosystems**

BMC Evolutionary Biology

Adiël A Klompmaker, Sten L. Jakobsen and Bodil W. Lauridsen

**Additional files**

**Additional file 1**

**
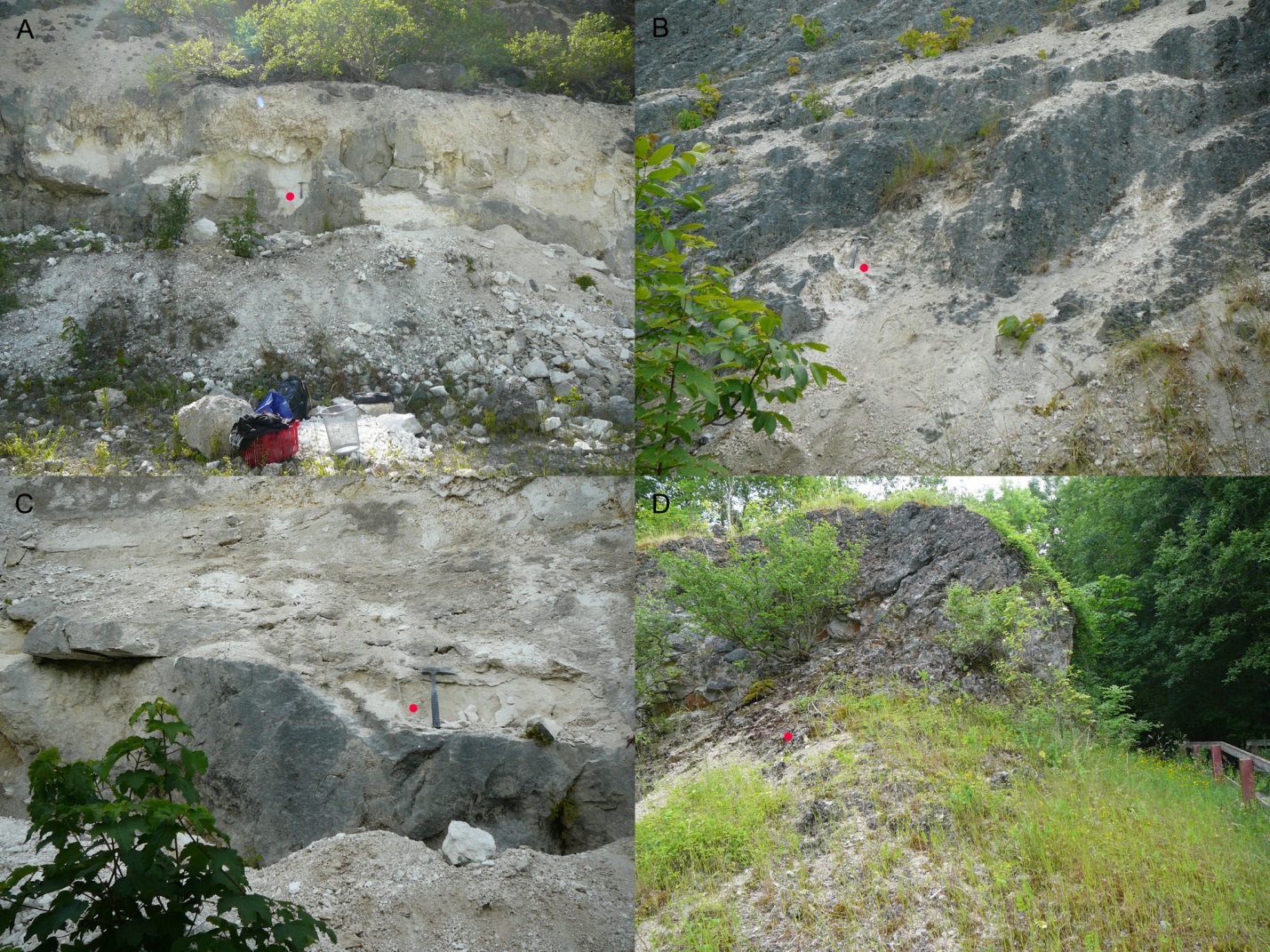
**

**Fig. S1** Images of the four study sites at the Faxe Quarry. A. Site 1. B. Site 2. C. Site 3. D. Site 4. The red dot (near the hammer in A–C) indicates the layer from which the buckets with sample material were collected.

**Table S1** Abundance data Faxe per taxon per sample without and with redistribution.

|  | without redistribution | | | | with redistribution | | | |
| --- | --- | --- | --- | --- | --- | --- | --- | --- |
|  | site 1 | site 2 | site 3 | site 4 | site 1 | site 2 | site 3 | site 4 |
| *Dromiopsis elegans* | 10 | 10 | 4 | 36 | 11 | 10 | 4 | 38 |
| *D. rugosus* | 5 | 11 | 0 | 16 | 5 | 11 | 0 | 17 |
| *D. ?laevior* | 0 | 0 | 0 | 1 | 0 | 0 | 0 | 1 |
| *Caloxanthus ornata* | 2 | 3 | 1 | 1 | 2 | 3 | 1 | 1 |
| *Titanocarcinus subellipticus* | 0 | 0 | 0 | 1 | 0 | 0 | 0 | 1 |
| *Faksecarcinus gracilis* | 0 | 0 | 0 | 2 | 0 | 0 | 0 | 2 |
| *Latheticocarcinus spinigus* | 0 | 0 | 0 | 4 | 0 | 0 | 0 | 5 |
| *Galathea strigifera* | 2 | 1 | 1 | 19 | 3 | 3 | 3 | 26 |
| *Protomunida munidoides* | 4 | 1 | 0 | 33 | 6 | 3 | 0 | 45 |
| *Munida primaeva* | 0 | 0 | 0 | 1 | 0 | 0 | 0 | 1 |
| *Faxegalathea platyspinosa* | 0 | 0 | 0 | 3 | 0 | 0 | 0 | 4 |
| *Dromiopsis* sp. | 1 | 0 | 0 | 3 |  |  |  |  |
| Galatheoidea | 3 | 4 | 2 | 20 |  |  |  |  |
| Decapoda | 0 | 2 | 1 | 0 |  |  |  |  |
| Homolidae | 0 | 0 | 0 | 1 |  |  |  |  |

**Table S2** Size data Faxe per sample and site and per specimen. NA means that the width could not be determined.

| (width in mm) | *Dromiopsis elegans* | *Dromiopsis rugosus* | *Galathea strigifera* | *Dromiopsis* sp. | *Protomunida munidoides* | Galatheoidea | *Caloxanthus ornata* | Decapoda | *Faxegalathea platyspinosa* | *Munida primaeva* | *Dromiopsis ?laevior* | Homolidae | *Titanocarcinus subellipticus* | *Faksecarcinus gracilis* | *Latheticocarcinus spinigus* |
| --- | --- | --- | --- | --- | --- | --- | --- | --- | --- | --- | --- | --- | --- | --- | --- |
| Sample 1a | 11.68 |  | 4.22 | 5.54 |  |  |  |  |  |  |  |  |  |  |  |
| Sample 1a | NA |  |  |  |  |  |  |  |  |  |  |  |  |  |  |
| Sample 1b | 10.04 | 3.97 |  |  | 2.5 | 5.34 | NA |  |  |  |  |  |  |  |  |
| Sample 1b | 5.47 | 14.08 |  |  | 2.24 | NA | 5.88 |  |  |  |  |  |  |  |  |
| Sample 1b | 11.2 |  |  |  |  |  |  |  |  |  |  |  |  |  |  |
| Sample 1c | 11.68 | 6.12 | 5.7 |  |  |  |  |  |  |  |  |  |  |  |  |
| Sample 1c | 13.38 | 17.84 |  |  |  | 3.75 |  |  |  |  |  |  |  |  |  |
| Sample 1d | NA | 8.32 |  |  | 4.64 |  |  |  |  |  |  |  |  |  |  |
| Sample 1d | 10.64 |  |  |  | 4 |  |  |  |  |  |  |  |  |  |  |
| Sample 1d | 8.31 |  |  |  |  |  |  |  |  |  |  |  |  |  |  |
| Sample 2a | NA | 7.69 |  |  | 4.96 | 4.3 | 7.4 | NA |  |  |  |  |  |  |  |
| Sample 2a | NA | 9.4 |  |  |  |  |  | NA |  |  |  |  |  |  |  |
| Sample 2a | 5.2 | 7.43 |  |  |  |  |  |  |  |  |  |  |  |  |  |
| Sample 2b | NA | 22.3 | 4.6 |  |  | 3.75 |  |  |  |  |  |  |  |  |  |
| Sample 2b |  |  |  |  |  | 3.66 | 6.42 |  |  |  |  |  |  |  |  |
| Sample 2c | 5.67 | 27.8 |  |  |  |  | 9.26 |  |  |  |  |  |  |  |  |
| Sample 2c | 7.98 | 12.52 |  |  |  |  |  |  |  |  |  |  |  |  |  |
| Sample 2c | 2.97 | 22.07 |  |  |  |  |  |  |  |  |  |  |  |  |  |
| Sample 2c | 4.3 | 6.84 |  |  |  |  |  |  |  |  |  |  |  |  |  |
| Sample 2c | 9.26 |  |  |  |  |  |  |  |  |  |  |  |  |  |  |
| Sample 2d | 11.4 | 22.88 |  |  |  | NA |  |  |  |  |  |  |  |  |  |
| Sample 2d |  | 22.22 |  |  |  |  |  |  |  |  |  |  |  |  |  |
| Sample 2d |  | 21.4 |  |  |  |  |  |  |  |  |  |  |  |  |  |
| Sample 3a | NA |  | 2.46 |  |  |  |  |  |  |  |  |  |  |  |  |
| Sample 3a |  |  |  |  |  | 2.73 |  |  |  |  |  |  |  |  |  |
| Sample 3b | 6.12 |  |  |  |  | 3.52 |  | 6.28 |  |  |  |  |  |  |  |
| Sample 3b | 7.59 |  |  |  |  |  |  |  |  |  |  |  |  |  |  |
| Sample 3b | 6.36 |  |  |  |  |  | 7.4 |  |  |  |  |  |  |  |  |
| Sample 3c |  |  |  |  |  |  |  |  |  |  |  |  |  |  |  |
| Sample 3d |  |  |  |  |  |  |  |  |  |  |  |  |  |  |  |
| Sample 4a | 5.81 | 4.89 | 4.68 |  | 2.95 | 4 | 3.37 |  |  | 3.79 |  | 2.5 | 8.57 | 7.38 | 6.16 |
| Sample 4a | 4.26 | 4.1 | 5.14 |  | 5.8 | 5.14 |  |  |  |  |  |  |  |  | NA |
| Sample 4a | NA | NA | 7.2 |  | 4.8 | 3.14 |  |  |  |  |  |  |  |  |  |
| Sample 4a | 6.14 | 9.63 | 4 |  | 2.27 | 1.7 |  |  |  |  |  |  |  |  |  |
| Sample 4a | 7.78 | 6 | 3.76 |  | 2.38 | 3.14 |  |  |  |  |  |  |  |  |  |
| Sample 4a | 8.2 |  | 3.2 |  | 4.33 | 4.7 |  |  |  |  |  |  |  |  |  |
| Sample 4a | 5.1 |  | 4.92 |  | 2.52 | 3.64 |  |  |  |  |  |  |  |  |  |
| Sample 4a | 6.86 |  | 5.6 |  | 2.13 | 2.5 |  |  |  |  |  |  |  |  |  |
| Sample 4a |  |  | 3.88 |  | 2.8 | 3.4 |  |  |  |  |  |  |  |  |  |
| Sample 4a |  |  | 3.74 |  | 3.86 | 3.8 |  |  |  |  |  |  |  |  |  |
| Sample 4a |  |  | 2.62 |  | 2.5 |  |  |  |  |  |  |  |  |  |  |
| Sample 4a |  |  |  |  | 3.9 |  |  |  |  |  |  |  |  |  |  |
| Sample 4a |  |  |  |  | 3.16 |  |  |  |  |  |  |  |  |  |  |
| Sample 4b | 9.1 | 24.7 | 1.7 | 15.8 | 5.6 | 3.4 |  |  | 3.78 |  |  |  |  |  |  |
| Sample 4b | 11.28 |  | 2.7 |  | 4.8 | 4.7 |  |  |  |  |  |  |  |  |  |
| Sample 4b | 8.26 |  | 3.6 |  | 2.54 | 3.8 |  |  |  |  |  |  |  |  |  |
| Sample 4b | 11.64 |  | 4.02 |  | 4.28 | 4.46 |  |  |  |  |  |  |  |  |  |
| Sample 4b | 9.6 |  |  |  | 4.2 |  |  |  |  |  |  |  |  |  |  |
| Sample 4c | 9.46 | 15 | 4.66 | NA | 4.4 | 3.3 |  |  |  |  |  |  |  | NA | 5.3 |
| Sample 4c | 8.88 | 11.24 | 4.39 |  | 4.93 |  |  |  |  |  |  |  |  |  |  |
| Sample 4c | 8.8 | 16.44 |  |  | 4.02 |  |  |  |  |  |  |  |  |  |  |
| Sample 4c | 12 |  |  |  | 2.2 |  |  |  |  |  |  |  |  |  |  |
| Sample 4c | 8 |  |  |  | 4.59 |  |  |  |  |  |  |  |  |  |  |
| Sample 4c | 7.46 |  |  |  | 3.7 |  |  |  |  |  |  |  |  |  |  |
| Sample 4c |  |  |  |  | 3.97 |  |  |  |  |  |  |  |  |  |  |
| Sample 4d | 7.76 | 4.24 | 3.91 | NA | 2.7 | 4.06 |  |  | 2.7 |  | 1.9 |  |  |  | NA |
| Sample 4d | 10.54 | 16.24 | 3.2 |  | 3.97 | 4.08 |  |  | 3.3 |  |  |  |  |  |  |
| Sample 4d | 13.29 | 5.54 | 3.14 |  | 3.96 | 4.12 |  |  |  |  |  |  |  |  |  |
| Sample 4d | 7.49 | 7.68 | 3.72 |  | 4.86 | 4.3 |  |  |  |  |  |  |  |  |  |
| Sample 4d | 11.32 | 8.64 |  |  | 4.04 | 3.84 |  |  |  |  |  |  |  |  |  |
| Sample 4d | 11.7 | 19.8 |  |  | 4.31 |  |  |  |  |  |  |  |  |  |  |
| Sample 4d | 5.58 | 7.46 |  |  | 2.84 |  |  |  |  |  |  |  |  |  |  |
| Sample 4d | 13.02 |  |  |  | 2.82 |  |  |  |  |  |  |  |  |  |  |
| Sample 4d | 10.41 |  |  |  |  |  |  |  |  |  |  |  |  |  |  |
| Sample 4d | 6.3 |  |  |  |  |  |  |  |  |  |  |  |  |  |  |
| Sample 4d | 13.02 |  |  |  |  |  |  |  |  |  |  |  |  |  |  |
| Sample 4d | 11.8 |  |  |  |  |  |  |  |  |  |  |  |  |  |  |
| Sample 4d | 13.6 |  |  |  |  |  |  |  |  |  |  |  |  |  |  |
| Sample 4d | 4.67 |  |  |  |  |  |  |  |  |  |  |  |  |  |  |
| Sample 4d | 12.61 |  |  |  |  |  |  |  |  |  |  |  |  |  |  |
| Sample 4d | NA |  |  |  |  |  |  |  |  |  |  |  |  |  |  |
| Sample 4d | 6.05 |  |  |  |  |  |  |  |  |  |  |  |  |  |  |

**Table S3** Maximum size data of decapods from the Faxe locality and the references from which these sizes were derived. Only decapods with size data were included.

| **Faxe, Paleocene (Danian), Denmark** | **Maximum length excl. rostrum (mm)** | **Maximum width (mm)** | **Geometric mean** | **Reference for size** |
| --- | --- | --- | --- | --- |
| *“Plagiophthalmus” depressus* | 9.4 | 7.0 | 8.1 | Jakobsen and Collins, 1997 |
| *Dromiopsis rugosa* | 28.3 | 30.0 | 29.1 | Polkowsky, 2014 |
| *Dromiopsis elegans* | 18.0 | 21.0 | 19.4 | Damholt et al., 2010 |
| *Dromiopsis minor* | 27.0 | 27.0 | 27.0 | Polkowsky, 2014 |
| *Dromiopsis laevior* | 35.1 | 37.0 | 36.0 | von Fischer-Benzon, 1866 |
| *Latheticocarcinus transiens* | 24.0 | 20.7 | 22.3 | Jakobsen and Collins, 1997 |
| *Latheticocarcinus spinigus* | 16.5 | 13.9 | 15.1 | Jakobsen and Collins, 1997 |
| *Latheticocarcinus affinis* | 23.0 | 18.0 | 20.3 | Jakobsen and Collins, 1997 |
| *Latheticocarcinus adelphinus* | 11.6 | 9.0 | 10.2 | Damholt et al., 2010 |
| *Raniliformis baltica* | 23.5 | 22.0 | 22.7 | Damholt et al., 2010 |
| *Necrocarcinus senonensis* | 38.0 | 40.0 | 39.0 | Segerberg, 1900 |
| *Faksecarcinus gracilis* | 8.3 | 13.6 | 10.6 | herein |
| *Cyclocorystes incertus* | 10.6 | 11.0 | 10.8 | Damholt et al., 2010 |
| *Jakobsenius cretaceus* | 27.4 | 34.7 | 30.8 | Jakobsen and Collins, 1997 |
| *Titanocarcinus subellipticus* | 18.2 | 21.0 | 19.5 | Jakobsen and Collins, 1997 |
| *Titanocarcinus faxeensis* | 10.5 | 10.0 | 10.2 | Damholt et al., 2010 |
| *Caloxanthus ornatus* | 10.1 | 13.8 | 11.8 | herein |
| *Faxegalathea platyspinosa* | 7.1 | 6.5 | 6.8 | Jakobsen and Collins, 1997 |
| *Protomunida minudoides* | 7.0 | 5.9 | 6.4 | Jakobsen and Collins, 1997 |
| *Galathea strigifera* | 4.5 | 3.8 | 4.1 | Jakobsen and Collins, 1997 |
| *Munida primaeva* | 5.6 | 4.5 | 5.0 | Jakobsen and Collins, 1997 |

**Table S4** Maximum size data of decapods from the ENCI/St Pietersberg locality and the references from which these sizes were derived. Only decapods with size data were included.

| **ENCI/St Pietersberg, Upper Cretaceous (Maastrichtian), The Netherlands** | **Maximum length excl. rostrum (mm)** | **Maximum width (mm)** | **Geometric mean** | **Reference for size** |
| --- | --- | --- | --- | --- |
| *Caloxanthus kuypersi* | 5.9 | 7.8 | 6.8 | Fraaye, 1996a |
| *Cosmonotus chevrona* | 10.3 | 8.1 | 9.1 | Fraaye and Van Bakel, 1998 |
| *Cretachlorodius enciensis* | 12.0 | 25.0 | 17.3 | Fraaye, 1996b |
| *Dromiopsis praelevior* | 12.0 | 13.2 | 12.6 | Collins et al., 1995 |
| *Glyptodynomene inornata* | 6.0 | 6.0 | 6.0 | Collins et al., 1995 |
| *Graptocarcinus maastrichtensis* | 8.0 | 11.0 | 9.4 | Fraaye, 1996a |
| *Latheticocarcinus declinatus* | 8.5 | 6.0 | 7.1 | Collins et al., 1995 |
| *Lyreidina pyriformis* | 12.9 | 8.2 | 10.3 | Fraaye and Van Bakel, 1998 |
| *Orithopsis angelicus* | 16.2 | 17.2 | 16.7 | Van Bakel et al., 2012 |
| *Paranecrocarcinus vanbirgeleni* | 18.0 | 20.0 | 19.0 | Fraaye, 1996b |
| *Raninella quadrispinosum* | 36.7 | 24.7 | 30.1 | Collins et al., 1995 |
| *Xanthosia semiornata* | 16.0 | 31.6 | 22.5 | Jagt et al., 1991 |
| *Xanthosioides delicata* | 11.0 | 22.0 | 15.6 | Fraaye, 1996c |
| *Paragalathea ubaghsi* | 18.3 | 16.0 | 17.1 | Collins et al., 1995 |
| *Eomunidopsis meerssensis* | 6.2 | 5.2 | 5.7 | Collins et al., 1995 |

**Table S5** Maximum size data of decapods from the Koskobilo locality and the references from which these sizes were derived. Only decapods with size data were included.

| **Koskobilo, mid-Cretaceous (Albian), Spain** | **Maximum length excl. rostrum (mm)** | **Maximum width (mm)** | **Geometric mean** | **Reference for size** |
| --- | --- | --- | --- | --- |
| *Acareprosopon bouvieri* | 5.4 | 4.4 | 4.9 | Klompmaker, 2013 |
| *Albenizus minutus* | 3.5 | 3.1 | 3.3 | Klompmaker, 2013 |
| *Caloxanthus paraornatus* | 5.0 | 6.6 | 5.7 | Klompmaker et al., 2013a |
| *Cretamaja granulata* | 15.2 | 11.3 | 13.1 | Klompmaker. 2013 |
| *Distefania incerta* | 40.0 | 48.5 | 44.0 | Klompmaker et al., 2013a |
| *Distefania renefraaijei* | 14.6 | 20.0 | 17.1 | Klompmaker et al., 2012b |
| *Eodromites grandis* | 14.0 | 13.6 | 13.8 | Klompmaker et al., 2012b |
| *Etyxanthosia fossa* | 8.0 | 10.2 | 9.0 | Klompmaker et al., 2011a |
| *Faksecarcinus koskobiloensis* | 11.0 | 20.1 | 14.9 | Klompmaker et al., 2013a |
| *Glyptodynomene alsasuensis* | 10.2 | 11.8 | 11.0 | Klompmaker et al., 2013a |
| *Goniodromites laevis* | 20.2 | 20.9 | 20.5 | Klompmaker et al., 2013a |
| *Graptocarcinus texanus* | 26.4 | 31.5 | 28.8 | Klompmaker, 2013 |
| *Koskobilius postangustus* |  | 4.8 |  | Klompmaker, 2013 |
| *Laeviprosopon crassum* | 14.8 | 13.3 | 14.0 | Klompmaker, 2013 |
| *Laeviprosopon edoi* | 8.0 | 4.9 | 6.3 | Klompmaker, 2013 |
| *Laeviprosopon hispanicum* | 16.3 | 13.3 | 14.7 | Klompmaker, 2013 |
| *Laeviprosopon planum* | 4.7 | 4.0 | 4.3 | Klompmaker, 2013 |
| *Navarradromites pedroartali* | 10.9 | 9.0 | 9.9 | Klompmaker et al., 2012b |
| *Navarrahomola hispanica* | 24.9 | 19.6 | 22.1 | Artal et al., 2012 |
| *Navarrara betsiei* | 5.5 | 4.9 | 5.2 | Klompmaker et al., 2013a |
| *Rathbunopon obesum* | 13.5 | 11.4 | 12.4 | Klompmaker et al., 2011c |
| *Viaia robusta* | 18.9 | 10.5 | 14.1 | Klompmaker et al., 2013a |
| *Mesoparapylocheles michaeljacksoni* | | 5.2 |  | Klompmaker et al., 2013a |
| *Annuntidiogenes worfi* |  | 2.9 |  | Fraaije et al., 2012 |
| *Cretatrizocheles olazagutiensis* | | 4.4 |  | Fraaije et al., 2012 |
| *Eomunidopsis aldoirarensis* | 8.3 | 6.8 | 7.5 | Klompmaker et al., 2012a |
| *Eomunidopsis navarrensis* | 12.5 | 11.3 | 11.9 | Klompmaker et al., 2012a |
| *Eomunidopsis orobensis* | 12.8 | 10.1 | 11.4 | Klompmaker et al., 2012a |
| *Gastrodorus cretahispanicus* | 6.6 | 3.5 | 4.8 | Klompmaker et al., 2013a |
| *Hispanigalathea pseudolaevis* | 4.0 | 3.3 | 3.6 | Klompmaker et al., 2012a |
| *Hispanigalathea tuberosa* | 5.9 | 4.6 | 5.2 | Klompmaker et al., 2012a |
| *Nykteripteryx rostrata* | 2.4 | 2.6 | 2.5 | Klompmaker et al., 2012a |
| *Paragalathea multisquamata* | 7.6 | 6.6 | 7.1 | Klompmaker et al., 2012a |
| *Paragalathea ruizi* | 7.1 | 6.1 | 6.6 | Klompmaker et al., 2013a |
| *Paragalathea straeleni* | 19.2 | 17.1 | 18.1 | Klompmaker et al., 2012a |

**Table S6** Maximum size data of decapods from Contrada Gecchelina di Monte di Malo and the references from which these sizes were derived. Only decapods with size data were included.

| **Contrada Gecchelina di Monte di Malo, Eocene (Ypresian), Italy** | **Maximum length (incl. front) (mm)** | **Maximum width (mm)** | **Geometric mean** | **Reference for size** |
| --- | --- | --- | --- | --- |
| *Cyamocarcinus angustifrons* |  | 39.3 |  | Beschin et al., 2007 |
| *?Diaulax italica* | 5.4 | 5.6 | 5.5 | Beschin et al., 2007 |
| *Dromiopsis paucigranosa* |  | 6.4 |  | Beschin et al., 2007 |
| *Eotrachynotocarcinus airaghii* | | 19.4 |  | Beschin et al., 2007 |
| *Kromtitis koberiformis* | 16.9 | 18.5 | 17.7 | Beschin et al., 2007 |
| *Kromtitis levigatus* |  | 12.2 |  | Beschin et al., 2007 |
| *Kromtitis subovatus* |  | 17.4 |  | Beschin et al., 2007 |
| *Guinotosia tertiaria* | 16.7 | 27.8 | 21.5 | Beschin et al., 2007 |
| *Daira sicula* |  | 26.4 |  | Beschin et al., 2007 |
| *Daldorfia eocaena* |  | 20.4 |  | Beschin et al., 2007 |
| *Mesolambrus declinatus* | 9.2 | 15.0 | 11.7 | Beschin et al., 2007 |
| *Branchioplax parva* | 7.0 | 9.4 | 8.1 | Beschin et al., 2007 |
| *Branchioplax sulcata* | 4.6 | 5.8 | 5.2 | Beschin et al., 2007 |
| *Boschettia giampietroi* | 7.8 | 8.6 | 8.2 | Beschin et al., 2007 |
| *Eocharybdis cristata* | 4.0 | 4.2 | 4.1 | Beschin et al., 2007 |
| *Gecchelicarcinus lorigae* | 18.6 | 24.0 | 21.1 | Beschin et al., 2007 |
| *Carpilius petreus* |  | 30.5 |  | Beschin et al., 2007 |
| *Paraocalina multilobata* | 14.4 | 22.5 | 18.0 | Beschin et al., 2007 |
| *Bittnereus vicentinus* |  | 55.4 |  | Beschin et al., 2007 |
| *Laevicarcinus lioyi* |  | 19.0 |  | Beschin et al., 2007 |
| *Panopeus incisus* |  | 17.4 |  | Beschin et al., 2007 |
| *Sereneopeus humilis* |  | 24.5 |  | Beschin et al., 2007 |
| *Galenopsis similis* |  | 61.3 |  | Beschin et al., 2007 |
| *Lobogalenopsis quadrilobata* |  | 20.8 |  | Beschin et al., 2007 |
| *Pilumnomimus* cf. *planidentatus* | | 11.0 |  | Beschin et al., 2007 |
| *Titanocarcinus raulinianus* |  | 28.3 |  | Beschin et al., 2007 |
| *Paratetralia convexa* |  | 14.3 |  | Beschin et al., 2007 |
| *Actaeites lobatus* | 13.8 | 17.2 | 15.4 | Beschin et al., 2007 |
| *Etisus arduinoi* | 16.7 | 22.7 | 19.5 | Beschin et al., 2007 |
| *Haydnella maladensis* | 11.3 | 14.4 | 12.8 | Beschin et al., 2007 |
| *Neoliomera paleogenica* | 19.3 | 31.2 | 24.5 | Beschin et al., 2007 |
| *Phlyctenodes multituberculatus* | | 31.0 |  | Beschin et al., 2007 |
| *Prochlorodius ellipticus* | 5.7 | 9.5 | 7.4 | Beschin et al., 2007 |
| *Eoplax minuscula* | 5.7 | 7.0 | 6.3 | Beschin et al., 2007 |
| *Brachynotus corallinus* | 8.7 | 10.0 | 9.3 | Beschin et al., 2007 |
| *Acanthogalathea squamosa* | 5.5 | 5.6 | 5.5 | Beschin et al., 2007 |
| *Lessinigalathea regale* |  | 13.2 |  | Beschin et al., 2007 |
| *Pachycheles dorsosulcatus* | 7.2 | 7.2 | 7.2 | Beschin et al., 2007 |

**Table S7** Maximum size data of decapods from the Braggi locality and the references from which these sizes were derived. Only decapods with size data were included.

| **Braggi quarry at Vestenanova, Eocene (Ypresian), Italy** | **Maximum length (incl. front) (mm)** | **Maximum width (mm)** | **Geometric mean** | **Reference for size** |
| --- | --- | --- | --- | --- |
| *Paradistefania piccolii* |  | 9.6 |  | Beschin et al., 2015 |
| *Dromiopsis paucigranosa* | 6.8 | 7.1 | 6.9 | Beschin et al., 2015 |
| *Kromtitis subovatus* | 10.7 | 10.7 | 10.7 | Beschin et al., 2015 |
| *Metadynomene veronensis* | 5.3 | 6.3 | 5.8 | Beschin et al., 2015 |
| *Cyamocarcinus angustifrons* |  | 73.6 |  | Beschin et al., 2015 |
| *Eotrachynotocarcinus airaghii* | | 19.9 |  | Beschin et al., 2015 |
| *Corallomursia eocaena* |  | 5.6 |  | Beschin et al., 2015 |
| *Corallomursia pauciornata* |  | 5.9 |  | Beschin et al., 2015 |
| *Mesolambrus declinatus* |  | 9.8 |  | Beschin et al., 2015 |
| *Mesolambrus ypresianus* |  | 10.3 |  | Beschin et al., 2015 |
| *Daira sicula* |  | 11.3 |  | Beschin et al., 2015 |
| *Daira vestenanovensis* |  | 23.9 |  | Beschin et al., 2015 |
| *Gecchelicarcinus lorigae* |  | 14.1 |  | Beschin et al., 2015 |
| *Vestenanovia carinata* |  | 31.0 |  | Beschin et al., 2015 |
| *Neptocarcinus dezanchei* |  | 13.4 |  | Beschin et al., 2015 |
| *Braggicarpilius marginatus* |  | 6.5 |  | Beschin et al., 2015 |
| *Carpilius petreus* |  | 34.8 |  | Beschin et al., 2015 |
| *Paraocalina multilobata* |  | 35.6 |  | Beschin et al., 2015 |
| *Galenopsis similis* |  | 29.4 |  | Beschin et al., 2015 |
| *Lobogalenopsis quadrilobata* |  | 9.4 |  | Beschin et al., 2015 |
| *Palladiocarcinus brevidentatus* | 11.6 | 16.0 | 13.6 | Beschin et al., 2015 |
| *Laevicarcinus lioyi* |  | 13.2 |  | Beschin et al., 2015 |
| *Paratetralia convexa* |  | 10.0 |  | Beschin et al., 2015 |
| *Haydnella maladensis* |  | 7.7 |  | Beschin et al., 2015 |
| *Neoliomera minuta* | 3.8 | 6.0 | 4.8 | Beschin et al., 2015 |
| *Phlyctenodes krenneri* | 6.9 | 10.4 | 8.5 | Beschin et al., 2015 |
| *Phlyctenodes multituberculatus* | | 10.5 |  | Beschin et al., 2015 |
| *Actaeites lobatus* | 8.0 | 10.1 | 9.0 | Beschin et al., 2015 |
| *Muelleroplax minuscula* |  | 6.4 |  | Beschin et al., 2015 |
| *Prochlorodius ellipticus* | 11.6 | 18.5 | 14.6 | Beschin et al., 2015 |
| *Chasmocarcinus* cf. *guerini* |  | 4.3 |  | Beschin et al., 2015 |
| *Branchioplax parva* |  | 6.2 |  | Beschin et al., 2015 |
| *Branchioplax sulcata* |  | 7.4 |  | Beschin et al., 2015 |
| *Acanthogalathea squamosa* |  | 4.4 |  | Beschin et al., 2015 |
| *Lessinigalathea regale* |  | 5.4 |  | Beschin et al., 2015 |

**Table S8** Carapace and eye socket size measurements of *Caloxanthus* and *Faksecarcinus*.

| **Species** | **Age** | **Locality** | **Maximum length excl. rostrum (mm)** | **Maximum width (mm)** | **Geometric mean** | **Maximum height eye socket (mm)** | **Source, Museum number if known** |
| --- | --- | --- | --- | --- | --- | --- | --- |
| *Caloxanthus paraornatus* | late Albian | Koskobilo, Spain | 3.7 | 4.62 | 4.13 | 0.82 | Klompmaker et al., 2011a, MAB k2649 |
| *C. paraornatus* | late Albian | Koskobilo, Spain | 3.94 | 5.27 | 4.56 | 0.92 | Klompmaker et al., 2011a, MGSB 77703 |
| *C. paraornatus* | late Albian | Koskobilo, Spain | 3.48 | 4.68 | 4.04 | 0.85 | herein, MAB k3557 |
| *C. paraornatus* | late Albian | Koskobilo, Spain | 3.16 | 4.23 | 3.66 | 0.70 | herein, MAB k3558 |
| *C. paraornatus* | late Albian | Koskobilo, Spain | 5.01 | 6.26 | 5.60 | 0.95 | herein, MAB k3559 |
| *C. paraornatus* | late Albian | Koskobilo, Spain | 3.79 | 5.06 | 4.38 | 0.85 | herein, MAB k3560 |
| *C. paraornatus* | late Albian | Koskobilo, Spain | 4.3 | 5.37 | 4.81 | 0.88 | herein, MAB k3561 |
| *C. paraornatus* | late Albian | Koskobilo, Spain |  | 6.32 |  | 0.95 | herein, MAB k3562 |
| *C. paraornatus* | late Albian | Koskobilo, Spain |  | 4.42 |  | 0.76 | herein, MAB k3563 |
| *C. paraornatus* | late Albian | Koskobilo, Spain | 4.3 | 5.69 | 4.95 | 0.88 | herein, MAB k3564 |
| *C. paraornatus* | late Albian | Koskobilo, Spain | 4.04 | 5.37 | 4.66 | 0.92 | herein, MAB k3565 |
| *C. paraornatus* | late Albian | Koskobilo, Spain | 3.48 | 4.42 | 3.92 | 0.79 | herein, MAB k3566 |
| *C. paraornatus* | late Albian | Koskobilo, Spain | 4.42 | 6 | 5.15 | 0.95 | herein |
| *C. paraornatus* | late Albian | Koskobilo, Spain | 3.54 | 4.61 | 4.04 | 0.73 | herein |
| *C. paraornatus* | late Albian | Koskobilo, Spain | 2.4 | 3.41 | 2.86 | 0.63 | herein |
| *C. paraornatus* | late Albian | Koskobilo, Spain | 3.79 | 5.18 | 4.43 | 0.85 | herein |
| *C. paraornatus* | late Albian | Koskobilo, Spain | 3.22 | 4.68 | 3.88 | 0.82 | herein |
| *C. paraornatus* | late Albian | Koskobilo, Spain | 3.48 | 4.8 | 4.09 | 0.79 | herein |
| *C. paraornatus* | late Albian | Koskobilo, Spain |  | 3.67 |  | 0.63 | herein |
| *C. paraornatus* | late Albian | Koskobilo, Spain | 3.35 | 4.8 | 4.01 | 0.82 | herein, Klompmaker et al., 2013a |
| *C. paraornatus* | late Albian | Koskobilo, Spain |  | 6 |  |  | Klompmaker et al., 2013a |
| *C. paraornatus* | late Albian | Koskobilo, Spain |  | 6.1 |  |  | Klompmaker et al., 2013a |
| *C. paraornatus* | late Albian | Koskobilo, Spain |  | 4.7 |  |  | Klompmaker et al., 2013a |
| *C. paraornatus* | late Albian | Koskobilo, Spain |  | 5.2 |  |  | Klompmaker et al., 2013a |
| *C. paraornatus* | late Albian | Koskobilo, Spain |  | 4.6 |  |  | Klompmaker et al., 2013a |
| *C. paraornatus* | late Albian | Koskobilo, Spain |  | 6.6 |  |  | Klompmaker et al., 2013a |
| *C. paraornatus* | late Albian | Koskobilo, Spain |  | 5.6 |  |  | Klompmaker et al., 2013a |
| *C. ornatus* | middle Danian | Faxe, Denmark | 9.03 | 11.88 | 10.36 | 1.66 | herein |
| *C. ornatus* | middle Danian | Faxe, Denmark | 4.07 | 5.85 | 4.88 | 1 | herein |
| *C. ornatus* | middle Danian | Faxe, Denmark | 5.34 | 6.97 | 6.10 | 1.3 | herein |
| *C. ornatus* | middle Danian | Faxe, Denmark | 5.11 | 6.68 | 5.84 | 1.08 | herein |
| *C. ornatus* | middle Danian | Faxe, Denmark | 3.4 | 4.41 | 3.87 | 0.78 | herein |
| *C. ornatus* | middle Danian | Faxe, Denmark | 5.58 | 7.02 | 6.26 | 1.21 | herein, MAB k1522 |
| *C. ornatus* | middle Danian | Faxe, Denmark | 4.58 | 5.89 | 5.19 | 1.12 | herein |
| *C. ornatus* | middle Danian | Faxe, Denmark | 5.21 | 6.8 | 5.95 | 1.23 | herein |
| *C. ornatus* | middle Danian | Faxe, Denmark | 6.24 | 8.3 | 7.20 | 1.35 | herein |
| *C. ornatus* | middle Danian | Faxe, Denmark | 6.65 | 8.41 | 7.48 | 1.45 | herein |
| *C. ornatus* | middle Danian | Faxe, Denmark | 7.2 | 9.75 | 8.38 | 1.5 | herein |
| *C. ornatus* | middle Danian | Faxe, Denmark | 6.9 | 8.8 | 7.79 | 1.49 | Klompmaker et al., 2011a, MAB k3153 |
| *C. ornatus* | middle Danian | Faxe, Denmark | 4 | 5.1 | 4.52 | 0.91 | Klompmaker et al., 2011a, MAB k3155 |
| *C. ornatus* | middle Danian | Faxe, Denmark | 5.4 | 7.1 | 6.19 | 1.17 | Klompmaker et al., 2011a, MAB k3156 |
| *C. ornatus* | middle Danian | Faxe, Denmark |  | 7.09 |  | 1.25 | herein, GM 1991 1838 |
| *C. ornatus* | middle Danian | Faxe, Denmark | 7.36 | 9.35 | 8.30 | 1.48 | herein, GM 1991 1330 |
| *C. ornatus* | middle Danian | Faxe, Denmark | 5.75 | 7.11 | 6.39 | 1.22 | herein, GM 1991 1264 |
| *C. ornatus* | middle Danian | Faxe, Denmark | 7.1 | 8.99 | 7.99 | 1.45 | herein |
| *C. ornatus* | middle Danian | Faxe, Denmark | 8.23 | 10.71 | 9.39 | 1.71 | herein |
| *C. ornatus* | middle Danian | Faxe, Denmark | 5.6 | 7.3 | 6.39 | 1.37 | herein |
| *C. ornatus* | middle Danian | Faxe, Denmark | 4.15 | 5.2 | 4.65 |  | herein |
| *C. ornatus* | middle Danian | Faxe, Denmark |  | 9.04 |  | 1.39 | herein |
| *C. ornatus* | middle Danian | Faxe, Denmark | 6.01 | 7.85 | 6.87 | 1.3 | herein |
| *C. ornatus* | middle Danian | Faxe, Denmark | 4.23 | 5.28 | 4.73 |  | herein |
| *C. ornatus* | middle Danian | Faxe, Denmark |  | 13.8 |  |  | herein |
| *C. ornatus* | middle Danian | Faxe, Denmark |  | 7.51 |  |  | herein |
| *C. ornatus* | middle Danian | Faxe, Denmark | 4.27 | 5.8 | 4.98 | 0.93 | herein |
| *C. ornatus* | middle Danian | Faxe, Denmark | 7.7 | 9.99 | 8.77 | 1.61 | herein |
| *C. ornatus* | middle Danian | Faxe, Denmark |  | 4.61 |  |  | herein |
| *C. ornatus* | middle Danian | Faxe, Denmark | 8.21 | 10.55 | 9.31 | 1.66 | herein |
| *C. ornatus* | middle Danian | Faxe, Denmark | 6.25 | 8.23 | 7.17 |  | herein |
| *C. ornatus* | middle Danian | Faxe, Denmark | 7.07 | 8.68 | 7.83 | 1.33 | herein |
| *C. ornatus* | middle Danian | Faxe, Denmark | 4.95 | 6.16 | 5.52 | 0.9 | herein |
| *C. ornatus* | middle Danian | Faxe, Denmark | 5.83 | 7.68 | 6.69 | 1.2 | herein |
| *C. ornatus* | middle Danian | Faxe, Denmark |  | 9.56 |  | 1.46 | herein |
| *C. ornatus* | middle Danian | Faxe, Denmark | 6.53 | 8.6 | 7.49 | 1.44 | herein |
| *C. ornatus* | middle Danian | Faxe, Denmark | 7.88 | 10.23 | 8.98 | 1.58 | herein |
| *C. ornatus* | middle Danian | Faxe, Denmark | 5.19 | 6.72 | 5.91 | 1.08 | herein |
| *C. ornatus* | middle Danian | Faxe, Denmark | 5.46 | 6.99 | 6.18 | 1.13 | herein |
| *C. ornatus* | middle Danian | Faxe, Denmark |  | 9.21 |  | 1.32 | herein, GM 1991 1249 |
| *C. ornatus* | middle Danian | Faxe, Denmark | 5.83 | 7.17 | 6.47 | 1.27 | herein, GM 1991 1263 |
| *C. ornatus* | middle Danian | Faxe, Denmark | 9.11 | 11.36 | 10.17 | 1.69 | herein, GM 1991 1266 |
| *C. ornatus* | middle Danian | Faxe, Denmark | 3.81 | 4.76 | 4.26 | 0.9 | herein, GM 1991 1251 |
| *C. ornatus* | middle Danian | Faxe, Denmark | 8.34 | 10.56 | 9.38 | 1.58 | herein, GM 1991 1268 |
| *C. ornatus* | middle Danian | Faxe, Denmark | 6.67 | 8.72 | 7.63 | 1.31 | herein, GM 1991 1247 |
| *C. ornatus* | middle Danian | Faxe, Denmark | 5.14 | 6.45 | 5.76 | 1.07 | herein, GM 1991 1269 |
| *C. ornatus* | middle Danian | Faxe, Denmark | 5.7 | 7.49 | 6.53 | 1.12 | herein, GM 1991 1259 |
| *C. ornatus* | middle Danian | Faxe, Denmark | 3.33 | 4.22 | 3.75 | 0.77 | herein |
| *C. ornatus* | middle Danian | Faxe, Denmark | 6 | 7.86 | 6.87 | 1.29 | herein |
| *C. ornatus* | middle Danian | Faxe, Denmark | 5.43 | 6.8 | 6.08 | 1.17 | herein, GM 1991 1261 |
| *C. ornatus* | middle Danian | Faxe, Denmark | 6.49 | 8.32 | 7.35 | 1.36 | herein, GM 1991 1311 |
| *C. ornatus* | middle Danian | Faxe, Denmark | 5.45 | 6.66 | 6.02 | 1 | herein, GM 1991 1294 |
| *C. ornatus* | middle Danian | Faxe, Denmark | 5.2 | 6.49 | 5.81 | 1.11 | herein, GM 1991 1295 |
| *C. ornatus* | middle Danian | Faxe, Denmark | 4.42 | 5.57 | 4.96 | 0.93 | herein, GM 1991 1265 |
| *C. ornatus* | middle Danian | Faxe, Denmark | 6.4 | 8.3 | 7.29 | 1.32 | herein, GM 1991 1298 |
| *C. ornatus* | middle Danian | Faxe, Denmark | 8.8 | 11.46 | 10.04 | 1.73 | herein, GM 1991 1285 |
| *C. ornatus* | middle Danian | Faxe, Denmark | 6.58 | 8.42 | 7.44 | 1.43 | herein, GM 1991 1275 |
| *C. ornatus* | middle Danian | Faxe, Denmark | 5.18 | 6.39 | 5.75 |  | herein, GM 1991 1274 |
| *C. ornatus* | middle Danian | Faxe, Denmark | 8.91 | 11.44 | 10.10 |  | herein, GM 1991 1255 |
| *C. ornatus* | middle Danian | Faxe, Denmark | 5.64 | 7.69 | 6.59 | 1.19 | herein, GM 1991 1288 |
| *C. ornatus* | middle Danian | Faxe, Denmark | 4.66 | 6.04 | 5.31 | 1.05 | herein, GM 1991 1270 |
| *C. ornatus* | middle Danian | Faxe, Denmark | 4.81 | 6.08 | 5.41 | 1.15 | herein, GM 1991 1272 |
| *C. ornatus* | middle Danian | Faxe, Denmark | 4.69 | 6.2 | 5.39 | 1.15 | herein, GM 1991 1331 |
| *C. ornatus* | middle Danian | Faxe, Denmark | 5.15 | 6.7 | 5.87 | 1.2 | herein, GM 1991 1305 |
| *C. ornatus* | middle Danian | Faxe, Denmark | 6.57 | 8.54 | 7.49 |  | herein, GM 1991 1279 |
| *C. ornatus* | middle Danian | Faxe, Denmark | 7.52 | 9.66 | 8.52 | 1.52 | herein |
| *C. ornatus* | middle Danian | Faxe, Denmark | 7.78 | 10.22 | 8.92 | 1.68 | herein |
| *C. ornatus* | middle Danian | Faxe, Denmark | 7.19 | 9.14 | 8.11 |  | herein |
| *C. ornatus* | middle Danian | Faxe, Denmark | 6.99 | 9.25 | 8.04 | 1.45 | herein |
| *C. ornatus* | middle Danian | Faxe, Denmark | 5.53 | 6.77 | 6.12 | 1.22 | herein |
| *C. ornatus* | middle Danian | Faxe, Denmark |  | 12.45 |  |  | herein, GM 1991 1329 |
| *C. ornatus* | middle Danian | Faxe, Denmark | 5.17 | 6.59 | 5.84 | 1.23 | herein, GM 1991 1319 |
| *C. ornatus* | middle Danian | Faxe, Denmark | 7.6 | 10.17 | 8.79 | 1.58 | herein, GM 1991 1318 |
| *C. ornatus* | middle Danian | Faxe, Denmark | 7.47 | 9.84 | 8.57 |  | herein, GM 1991 1332 |
| *C. ornatus* | middle Danian | Faxe, Denmark | 5.24 | 7 | 6.06 |  | herein, GM 1991 1320 |
| *C. ornatus* | middle Danian | Faxe, Denmark | 6.22 | 8.1 | 7.10 | 1.27 | herein, GM 1991 1307 |
| *C. ornatus* | middle Danian | Faxe, Denmark | 4.79 | 5.94 | 5.33 | 0.98 | herein, GM 1991 1291 |
| *C. ornatus* | middle Danian | Faxe, Denmark | 4.9 | 6.32 | 5.56 | 1.16 | herein, GM 1991 1326 |
| *C. ornatus* | middle Danian | Faxe, Denmark | 7.19 | 9.27 | 8.16 |  | herein, GM 1991 1324 |
| *C. ornatus* | middle Danian | Faxe, Denmark | 4.35 | 5.43 | 4.86 | 1.04 | herein, GM 1991 1328 |
| *C. ornatus* | middle Danian | Faxe, Denmark | 6.19 | 8.08 | 7.07 |  | herein, GM 1991 1308 |
| *C. ornatus* | middle Danian | Faxe, Denmark | 6.22 | 8.15 | 7.12 |  | herein, GM 1991 1327 |
| *C. ornatus* | middle Danian | Faxe, Denmark | 4.74 | 6.45 | 5.53 | 1.2 | herein |
| *C. ornatus* | middle Danian | Faxe, Denmark | 2.59 | 3.35 | 2.95 | 0.695 | herein |
| *C. ornatus* | middle Danian | Faxe, Denmark | 3.76 | 4.89 | 4.29 | 0.9 | herein |
| *C. vignyensis* | middle to upper Danian | Vigny, France | 5.1 | 6.5 | 5.76 | 0.76 | Klompmaker et al., 2015, SNSB-BSPG 1988 III 41 |
| *Faksecarcinus koskobiloensis* | late Albian | Koskobilo, Spain |  | 5.94 |  |  | Klompmaker et al., 2011a, MAB k2572A |
| *F. koskobiloensis* | late Albian | Koskobilo, Spain |  | 13.42 |  |  | Klompmaker et al., 2011a, MAB k3145 |
| *F. koskobiloensis* | late Albian | Koskobilo, Spain |  | 14.65 |  |  | Klompmaker et al., 2011a, MAB k3144 |
| *F. koskobiloensis* | late Albian | Koskobilo, Spain |  | 14.87 |  |  | Klompmaker et al., 2011a, MAB k2520 |
| *F. koskobiloensis* | late Albian | Koskobilo, Spain | 9.35 | 17.07 | 12.63 |  | Klompmaker et al., 2011a, MGSB 77702 |
| *F. koskobiloensis* | late Albian | Koskobilo, Spain |  | 17.47 |  |  | Klompmaker et al., 2011a, MAB k2518 |
| *F.* cf. *F. koskobiloensis* | late Albian | Koskobilo, Spain | 4.96 | 8.3 | 6.42 |  | Klompmaker et al., 2011a, MAB k2563A |
| *F.* cf. *F. koskobiloensis* | late Albian | Koskobilo, Spain |  | 13.44 |  |  | Klompmaker et al., 2011a, MAB k3149 |
| *F.* cf. *F. koskobiloensis* | late Albian | Koskobilo, Spain | 4.38 | 6.75 | 5.44 |  | Klompmaker et al., 2011a, MAB k3101 |
| *F. gracilis* | middle Danian | Faxe, Denmark | > 6.01 | 10.44 |  | 1.22 | herein, MGUH 24367 |
| *F. gracilis* | middle Danian | Faxe, Denmark | 8.26 | 13.56 | 10.58 |  | herein, MGUH 24368 |
| *F. gracilis* | middle Danian | Faxe, Denmark | 6.97 | 11.41 | 8.92 |  | herein, GM 1996 61 |
| *F. gracilis* | middle Danian | Faxe, Denmark | 8.26 | 12.9 | 10.32 | 1.62 | herein, GM 1996 65 |
| *F. gracilis* | middle Danian | Faxe, Denmark | 6.86 | 10.86 | 8.63 |  | herein, GM 1996 55 |
| *F. gracilis* | middle Danian | Faxe, Denmark | 2.84 | 4.07 | 3.40 |  | herein, GM 1996 53 |
| *F. gracilis* | middle Danian | Faxe, Denmark | 5.89 | 8.93 | 7.25 |  | herein, GM 1996 51 |
| *F. gracilis* | middle Danian | Faxe, Denmark |  | 7.72 |  |  | herein, GM 1996 54 |
| *F. gracilis* | middle Danian | Faxe, Denmark |  | 10.54 |  |  | herein, GM 1996 52 |
| *F. gracilis* | middle Danian | Faxe, Denmark | 6.74 | 11.08 | 8.64 |  | herein, GM 1996 58 |
| *F. gracilis* | middle Danian | Faxe, Denmark |  | 12.48 |  |  | herein, GM 1996 57 |
| *F. gracilis* | middle Danian | Faxe, Denmark | 6.85 | 10.99 | 8.68 | 1.3 | herein, OESM-10059-20282 |
| *F. gracilis* | middle Danian | Faxe, Denmark | 6.75 | 10.76 | 8.52 |  | herein, OESM-10059-21090 |
| *F. gracilis* | middle Danian | Faxe, Denmark | 8.51 | 12.58 | 10.35 |  | herein, OESM-10059-21091 |
| *F. gracilis* | middle Danian | Faxe, Denmark |  | 7.82 |  |  | herein, OESM-10059-21092 |
| *F. gracilis* | middle Danian | Faxe, Denmark | 5.4 | 8.25 | 6.67 | 1.14 | herein, OESM-10059-21093 |

**Table S9** Maximum size data of all Paleocene crabs. Taxa in bold are have been found in Faxe; those with a star have been confirmed from the Danian.

| **Paleocene Brachyura** | **Maximum carapace length excl. rostrum (mm)** | **Maximum carapace width (mm)** | **Geometric mean** | **Reference for size** |
| --- | --- | --- | --- | --- |
| **Trechmannius circularis* | 8.0 | 9.2 | 8.6 | Collins and Donovan, 2006 |
| *Dromiopsis americana* | 14.0 | 13.2 | 13.6 | Roberts, 1956 |
| ******* *“****Plagiophthalmus”*** ***depressus*** | **9.4** | **7.0** | **8.1** | **Jakobsen and Collins, 1997** |
| ****Dromiopsis* *elegans*** | **18.0** | **21.0** | **19.4** | **Damholt et al., 2010** |
| ****Dromiopsis laevior*** | **35.1** | **37.0** | **36.0** | **von Fischer-Benzon 1866** |
| **Dromiopsis* aff. *D. mosae* |  | 34.3 |  | Jagt et al., 2014 |
| ****Dromiopsis minor*** | **27.0** | **27.0** | **27.0** | **Polkowsky, 2014** |
| ****Dromiopsis rugosus*** | **28.3** | **30.0** | **29.1** | **Polkowsky, 2014** |
| **Kierionopsis nodosa* | 23.6 | 23.0 | 23.3 | Davidson, 1966 |
| **Dromilites? cardwelli* | 12.1 | 12.6 | 12.3 | Schweitzer and Feldmann, 2012 |
| **Dromilites americana* | 18.4 | 18.3 | 18.3 | Rathbun, 1935 |
| **Kromtitis daniensis* | (incomplete carapace) | |  |  |
| ****Latheticocarcinus adelphinus*** | **11.6** | **9.0** | **10.2** | **Damholt et al., 2010** |
| ****Latheticocarcinus affinis*** | **23.0** | **18.0** | **20.3** | **Jakobsen and Collins, 1997** |
| ****Latheticocarcinus spinigus*** | **16.5** | **13.9** | **15.1** | **Jakobsen and Collins, 1997** |
| ****Latheticocarcinus transiens*** | **24.0** | **20.7** | **22.3** | **Jakobsen and Collins, 1997** |
| ****Caloxanthus ornatus*** | **10.1** | **13.8** | **11.8** | **herein** |
| **Caloxanthus vignyensis* | 5.1 | 6.5 | 5.8 | Klompmaker et al., 2015 |
| *Camarocarcinus arnesoni* | 45.2 | 42.8 | 44.0 | Holland and Cvancara, 1958 |
| *Camarocarcinus obtusus* | 34.0 | 36.3 | 35.1 | Jakobsen and Collins, 1979 |
| *Camarocarcinus quinquetuberculatus* | 36.0 | 36.0 | 36.0 | Collins and Rasmussen, 1992 |
| *Macroacaena* *bispinulata* | 19.0 | 11.5 | 14.8 | Collins and Rasmussen, 1992 |
| *Macroacaena* *venturai* | 42.0 | 25.0 | 32.4 | Vega et al., 2007 |
| **Giulianolyreidus johnsoni* | 23.2 | 14.4 | 18.3 | Rathbun, 1935 |
| **Giulianolyreidus* *bidentatus* | | 7.6 |  | Rathbun, 1935 |
| *Rogueus* *robustus* | 30.0 | 20.0 | 24.5 | Collins and Jakobsen, 1995 |
| **Symethoides monmouthorum* | 16.7 | 9.9 | 12.9 | Van Bakel et al., 2012 |
| **Ranina libyca* | 17.0 | 12.0 | 14.3 | Van Straelen, 1935 |
| *?Ranina burleighensis* | (cheliped only) | |  |  |
| **Quasilaeviranina eocenica* | 18.1 | 12.5 | 15.0 | Rathbun, 1935 |
| **Quasilaeviranina ovalis* | 27.5 | 20.0 | 23.5 | Rathbun, 1935 |
| *Raninoides borealis* | 31.0 | 20.0 | 24.9 | Collins and Rasmussen, 1992 |
| **Raninoides treldenaesensis* | 27.0 | 15.4 | 20.4 | Armstrong et al., 2009 |
| ****Raniliformis baltica*** | **23.5** | **22.0** | **22.7** | **Damholt et al., 2010** |
| **Notosceles* *bournei* | 16.8 | 10.1 | 13.0 | Rathbun, 1928 |
| **Cristella hastata* | 10.2 | 11.9 | 11.0 | Collins and Rasmussen, 1992 |
| ****Necrocarcinus senonensis (=N. insignis)*** | **38.0** | **40.0** | **39.0** | **Segerberg, 1900** |
| **Campylostoma bispinosus* | 49.0 | 30.0 | 38.3 | Segerberg, 1900 |
| **Matutites americanus* |  | 11.6 |  | Rathbun, 1935 |
| *Archaeopus* *schenki* | 16.0 | 22.5 | 19.0 | Van Straelen, 1939 |
| **Costacopluma* *australis* | 14.4 | 15.5 | 14.9 | Feldmann et al., 1995 |
| *Costacopluma* *bifida* | 7.0 | 7.5 | 7.2 | Collins et al., 1994 |
| **Costacopluma* *grayi* | 9.6 | 11.4 | 10.5 | Feldmann et al., 2014 |
| **Costacopluma* *nordestina* | 14.5 | 15.7 | 15.1 | Feldmann and Martins Neto, 1995 |
| **Costacopluma* *salamanca* | 15.0 | 17.6 | 16.2 | Feldmann et al., 1997 |
| *Costacopluma* *senegalensis* | 8.0 | 7.0 | 7.5 | Rémy in Gorodiski and Rémy, 1959 |
| **Costacopluma* *squiresi* | 13.0 | 15.8 | 14.3 | Nyborg et al., 2009 |
| **Costacopluma* *texana* | 10.9 | 11.8 | 11.3 | Armstrong et al., 2009 |
| **Proterocarcinus lophos* | 23.7 | 35.0 | 28.8 | Feldmann et al., 1995 |
| **Coeloma* *macrodactylus* | 40.0 | 46.0 | 42.9 | Van Straelen, 1925 |
| *Coeloma* *martinezensis* | 32.0 | 42.5 | 36.9 | Rathbun, 1926 |
| ****Faksecarcinus gracilis*** | **8.3** | **13.6** | **10.6** | **herein** |
| *Portufuria enigmatica* | 12.7 | 15.3 | 13.9 | Collins et al., 2005 |
| *Proxicarpilius planifrons* | 28.5 | 43.0 | 35.0 | Collins and Morris, 1978 |
| ****Jakobsenius cretaceus*** | **27.4** | **34.7** | **30.8** | **Jakobsen and Collins, 1997** |
| **Paraverrucoides* *alabamensis* | 17.6 | 24.8 | 20.9 | Rathbun, 1935 |
| *Verrucoides verrucoides* | 16.5 | 25.0 | 20.3 | Collins and Rasmussen, 1992 |
| **Rocacarcinus gerthi* | 43.0 | 60.0 | 50.8 | Glaessner, 1930 |
| **Lobulata* *lobulata* | (Danian sizes not given) | |  |  |
| ****Cyclocorystes incertus*** | **10.6** | **11.0** | **10.8** | **Damholt et al., 2010** |
| **Lobonotus sturgeoni* | 16.0 | 12.0 | 13.9 | Tavora et al., 2005 |
| ****Titanocarcinus faxeensis*** | **10.5** | **10.0** | **10.2** | **Damholt et al., 2010** |
| **Titanocarcinus* *kambuhelensis* | 8.0 | 9.7 | 8.8 | Verhoff et al., 2009 |
| ****Titanocarcinus subellipticus*** | **18.2** | **21.0** | **19.5** | **Jakobsen and Collins, 1997** |
| *Zanthopsis jacobi* | 47.0 | 59.0 | 52.7 | Van Straelen, 1925 |
| **?Titanocarcinus polonicus* |  | 16.0 |  | Fraaye, 1994 |
| *“Xanthilites” traubi* | (incomplete carapace) | |  |  |
| *“Titanocarcinus” reisi* | 15.7 | 18.5 | 17.0 | Förster, 1970 |
| **Galenopsis* *americana* | (propodus) |  |  |  |
| *Glyphithyreus wetherelli* | 26.5 | 34.2 | 30.1 | Collins and Morris, 1978 |
| **Panopeus estellensis* | (dactyli) |  |  |  |
| *Panopeus jerseyensis* | (fixed finger) |  |  |  |
| *Pakicarcinus orientalis* | 10.0 | 12.5 | 11.2 | Collins and Morris, 1978 |
| **Palaeopinnixa rocaensis* | 8.8 | 12.6 | 10.5 | Feldmann et al., 1995 |
| **Stevea martini* | 5.3 | 11.1 | 7.7 | Feldmann et al., 2014 |
| *Branchioplax* *ballingi* | 7.0 | 8.0 | 7.5 | Remy in Remy and Tessier, 1954 |
| **Tehuacana tehuacana* | 14.5 | 15.1 | 14.8 | Stenzel, 1944 |
| **Chirinocarcinus* *wichmanni* | 12.7 | 14.7 | 13.7 | Feldmann et al., 1995 |
| *Orbitoplax aldersoni* | 10.4 | 15.2 | 12.6 | Squires, 1980 |
| **Viapinnixa mexiaensis* | 8.5 | 13.5 | 10.7 | Armstrong et al., 2009 |
| *Viapinnixa nodosa* | 9.6 | 15.0 | 12.0 | Collins and Rasmussen, 1992 |
| *Viapinnixa perrilliatae* | 15.9 | 23.9 | 19.5 | Vega et al., 2007 |

**Table S10** Standing global diversity of decapods and Brachyura or true crabs across the Cretaceous/Paleogene boundary. Diversity is shown at the species- and genus-levels. Standing global diversity of decapods and a subset (brachyurans or true crabs) across the Cretaceous-Paleogene boundary at the species- and genus-levels. Four columns are used to compare the number of taxa present in the Maastrichtian and the Danian: raw, standardized per 5 my, standardized per 100 marine formations globally as a proxy for outcrop area, and standardized per 100 maps with marine outcrops in Western Europe as a proxy for outcrop area (see Smith and McGowan, 2007).

|  | **raw** | **per 5 My** | **per 100 formations** | **per 100 maps with outcrop** |
| --- | --- | --- | --- | --- |
| Maastrichtian (decapod species) | 120 | 98 | 65 | 140 |
| Danian (decapod species) | 92 | 105 | 123 | 174 |
| Maastrichtian (decapod genera) | 75 | 61 | 40 | 87 |
| Danian (decapod genera) | 55 | 63 | 73 | 104 |
| Maastrichtian (brachyuran species) | 75 | 61 | 40 | 87 |
| Danian (brachyuran species) | 55 | 63 | 73 | 104 |
| Maastrichtian (brachyuran genera) | 51 | 42 | 27 | 59 |
| Danian (brachyuran genera) | 36 | 41 | 48 | 68 |

**Table S11** Data used to obtain the values in the last three columns of Table S10.

|  | **Duration in my (ICS 2015/01)** | **Number of marine formations (PBDB)** | **Number of maps with outcrop in Western Europe (from Smith and McGowan, 2007)** |
| --- | --- | --- | --- |
| Maastrichtian | 6.1 | 186 | 86 |
| Danian | 4.4 | 75 | 53 |
